# Geminivirus Activates *ASYMMETRIC LEAVES 2* to Accelerate Cytoplasmic DCP2-Mediated mRNA Turnover and Weakens RNA Silencing in *Arabidopsis*

**DOI:** 10.1371/journal.ppat.1005196

**Published:** 2015-10-02

**Authors:** Jian Ye, Junyi Yang, Yanwei Sun, Pingzhi Zhao, Shiqiang Gao, Choonkyun Jung, Jing Qu, Rongxiang Fang, Nam-Hai Chua

**Affiliations:** 1 Temasek Life Sciences Laboratory, National University of Singapore, Singapore; 2 State Key laboratory of Plant Genomics, Institute of Microbiology, Chinese Academy of Sciences, Beijing, China; 3 Laboratory of Plant Molecular Biology, Rockefeller University, New York, New York, United States of America; 4 Institute of Biochemistry, National Chung Hsing University, Taichung, Taiwan; The Ohio State University, UNITED STATES

## Abstract

Aberrant viral RNAs produced in infected plant cells serve as templates for the synthesis of dsRNAs. The derived virus-related small interfering RNAs (siRNA) mediate cleavage of viral RNAs by post-transcriptional gene silencing (PTGS), thus blocking virus multiplication. Here, we identified ASYMMETRIC LEAVES2 (AS2) as a new component of plant P body complex which mediates mRNA decapping and degradation. We found that AS2 promotes DCP2 decapping activity, accelerates mRNA turnover rate, inhibits siRNA accumulation and functions as an endogenous suppressor of PTGS. Consistent with these findings, *as2* mutant plants are resistant to virus infection whereas AS2 over-expression plants are hypersensitive. The geminivirus nuclear shuttle protein BV1 protein, which shuttles between nuclei and cytoplasm, induces AS2 expression, causes nuclear exit of AS2 to activate DCP2 decapping activity and renders infected plants more sensitive to viruses. These principles of gene induction and shuttling of induced proteins to promote mRNA decapping in the cytosol may be used by viral pathogens to weaken antiviral defenses in host plants.

## Author Summary

In higher plants, aberrant RNAs generated during virus replication serve as templates to make small interfering RNAs. These small RNAs are used by host as a defense mechanism to cleave viral RNAs thereby blocking virus replication. The anti-virus defense is attenuated by the host cellular mRNA turnover machinery which clears aberrant RNAs. Viruses may use encoded component(s) to activate host cellular mRNA turnover for their own benefits. In this study, we identified ASYMMETRIC LEAVES2 (AS2) as an activator of mRNA decapping and degradation and an endogenous suppressor of virus silencing. We showed that the geminivirus BV1 protein induces AS2 expression, causes nuclear exit of AS2 to activate mRNA decapping activity and renders infected plants more sensitive to viruses. Similar mechanisms may be used by other viral pathogens to weaken antiviral defenses in host plants and also mammals.

## Introduction

Host-virus interactions entail susceptibility and resistance mechanisms embedded in anti-pathogen strategies of hosts and anti-host resistance strategies mounted by successful viral pathogens. As obligate intracellular parasites viruses have evolved various strategies to hijack host components for their own survival and multiplication in hosts [1].

In higher plants, post-transcriptional gene silencing (PTGS) is an important native antiviral resistance machinery that suppresses viral gene expression through (si)RNA-directed viral RNA cleavage. Aberrant viral RNAs synthesized during replication of RNA viruses or transcribed from DNA viruses serve as a template for RNA-DEPENDENT RNA POLYMERASE6 (RDR6) to synthesize complementary RNAs [1]. The double stranded RNAs (dsRNAs) produced are processed by the plant’s small RNA pathway to generate siRNAs which mediate viral mRNA cleavage and silencing viral gene expression. The importance of the PTGS pathway in plant viral defense has elicited counter defense measures from the viral pathogens to overcome it. Plant viruses have evolved various viral RNA silencing repressors (VSR) to target different PTGS pathway components. For example, the *Tomato bushy stunt virus* P19 protein binds to siRNAs directly whereas the *Cucumber mosaic virus* 2b protein blocks the RNA cleavage activity of Argonaute1 (AGO1) in the RNA-induced Silencing Complex [2,3]. In addition to the VSRs, plants also encode endogenous silencing repressor such as the *RDR1* from *Nicotiana tabacum* to target the dsRNA biogenesis step [4].

Aberrant viral RNAs serve as templates for synthesis of dsRNAs by RDR6, and like cellular mRNAs they can be cleared by the host RNA degradation machinery [1]. The clearance of aberrant viral RNAs will deplete substrates for dsRNA synthesis and siRNA production thus attenuating PTGS. Degradation of cytoplasmic RNAs occurs in P-bodies which are conserved and dynamic protein-RNA aggregates. Among protein subunits of the P-body complex investigated so far, DCP2 is the only protein subunit that possesses enzymatic activity for removing 5' cap structure (m^7^GpppX-) *in vitro* [5]. The 5' cap structure is an essential feature of eukaryotic mRNA required for mRNA stability and efficient translation [6]. Plant mutants deficient in P-body components display severe postembryonic developmental defects, suggesting that these cytoplasmic bodies play important roles in regulating gene expression in plant developmental process [7]. Furthermore, plant DCP1 and DCP5 have been shown to be translation suppressors [7,8]. Other than DCP1, DCP2 and DCP5, similar phenotypic and functional co-localization analyses suggest that 3 additional factors VARICOSE (VCS), XRN4, and DHH1 are also key components of plant P-bodies [7]. Genetic and biochemical analysis have shown that mutations in *XRN4* [9] or *DCP2* [10] enhance PTGS presumably by increasing aberrant RNA levels.

In contrast to higher plants, the role of mRNA decapping in animal host defense against viruses is unclear. RNA viruses, including negative stranded RNA viruses and ambiviruses (the *Orthomyxoviridae*, *Bunyaviridae*, and *Arenaviridae* families), and dsRNA virus totivirus L-A, which infects *Saccharomyces cerevisiae*, provide their mRNAs with a 5′ cap structure via a cap-snatching mechanism. In this mechanism, the viral polymerase cleaves host mRNAs 10–13 nucleotides from the 5′ end and utilizes the capped fragment as a primer to synthesize viral transcripts [11]. Therefore, the decapping machinery of animal cells may act as an important immune system to accelerate turning over of viral mRNAs thereby limiting virus multiplication for this subgroup RNA viral pathogens [11,12]. On the contrary, the decapping machinery in higher plants is used to suppress PTGS thereby promoting virus replication [10]. Little is known about the roles of plant mRNA decapping on virus-plant interaction and virus pathogenesis, possibly because deficiencies in most of the genes encoding mRNA decapping machinery cause postembryonic lethality [7].

Geminiviruses are the largest group of plant DNA viruses whose compact genomes consist of small single-stranded DNA circles of 2–3 kb. Despite their small size, these viruses inflict big damages on many commercially important crops [13]. Owing to their small genome size the encoded genetic information is extremely compact. Among geminiviruses, members of the genus *Begomovirus*, such as *India cassava mosaic virus* (ICMV), possess two genomic components, DNA-A and DNA-B [14]. The DNA-A which encodes 5 gene products is involved in virus replication, transcriptional activation of viral genes and encapsulation of the viral genome. The DNA-B component encodes two proteins that are expressed at the late stage of virus infection, the nuclear shuttle protein BV1 (NSP) and the movement protein BC1 (MP), both being required for viral systemic movement. It is known that BV1 facilitates the intracellular trafficking of viral DNA between nuclei and cytoplasm, whereas BC1 potentiates cell-to-cell movement of viral DNA. BV1 is a virulence factor, found to suppress trans-membrane receptor kinase activity *in vitro* [15]. AC2 which is encoded by the DNA-A component has been reported as a VSR and it trans-activates host and viral genes [16].

We have previously identified a pathogenesis factor βC1 of the monopartite geminivirus TYLCCNV (*Tomato yellow leaf curl China virus*) that interacts with *Arabidopsis* ASYMMETRIC LEAVES 1 (AS1) to cause alterations in leaf development resulting in the manifestation of disease symptoms [17]. AS1 is needed for βC1 function as changes in leaf morphology elicited by this viral factor is largely attenuated in *as1* mutant. Unexpectedly, βC1 is able to partially complement *as2* mutation suggesting that βC1 is a molecular mimic of ASYMMETRIC LEAVES 2 (AS2) [17]. What roles does AS2 play in viral pathogenicity and/or virulence remains to be investigated.

Here, we show that BV1, a geminivirus virulence factor that is expressed late in the virus life cycle, can induce *AS2* expression by binding to its promoter region. Over-expression of AS2 both in *Arabidopsis thaliana* and *Nicotiana benthamiana* rendered plants more sensitive to geminivirus infection whereas *as2* mutant plants were resistant. Moreover, BV1 can induce nuclear export of AS2 to the cytosol where the latter interacted with DCP2 to promote decapping activity, reduce siRNA accumulation and weaken RNA silencing. This viral counter-defense strategy makes plants more sensitive to virus infection and replication. Finally, we provide evidence that cytoplasmic localization of AS2 was necessary for it to function as a negative regulator of host resistance against virus infection.

## Materials and Methods

### Plant materials, growth conditions, and transformation

*Arabidopsis thaliana* Wild type (WT) and *as1-1*, *as2-1*, and *sgs3-1*mutants (all in Col-0 background) were used [18–21]. After vernalization for 2 days at 4°C in darkness, seeds were germinated on Murashing and Skoog (MS) medium at 22°C with 16 h light. Plasmids were introduced into *Agrobacterium tumefaciens* strain AGL1 or EHA105 by electrotransformation. *Arabidopsis* transformations were performed using the floral-dip method [22].

*Nicotiana benthamiana* GFP transgene line 16c was kindly provided by Dr. David Baulcombe. *Agrobacterium*-mediated transient expression in *N*.*benthamiana* leaves was performed by pressure infiltration [23]. *Arabidopsis thaliana* L1 line with silenced *35S-GUS* transgene was from Dr HervéVaucheret [24].

Plants were infected with CaLCuV by either micro-particle bombardment or agroinfiltration. Plasmids pCPCbLCVA.007 (GenBank accession no.AY279345) and pCPCbLCVB.002 (GenBank accession no. AY279344) were kindly provided by Dr Dominique Robertson (Turnage et al., 2002). Biolistic PDS-1000/HE System (Biorad) was used for particle delivery and *Arabidopsis* plants were inoculated at 1,100 psi Capture Disks (Biorad). Inoculated plants were kept in darkness for 12h and then returned to a growth chamber under normal condition. Symptoms of the inoculated plants were recorded 7 days later. For quantitative analysis of viral titer, leaf samples were collected from plants 3–4 weeks after inoculation. Total gDNA and viral DNAs were extracted by CTAB method and quantitative PCR (Q-PCR) was performed by using *Actin2* as an internal genomic DNA control [25]. CaLCuV infectious clones (DNA-A and DNA-B) suitable for agroinfiltration [25] were used to infect *Arabidopsis* plants with 6–8 true leaves.

### Constructs

Full-length cDNAs were amplified by PCR using Phusion High-Fidelity DNA Polymerase (FINNZYMES) and subcloned into binary vectors pCAMBIA1300-2X35-3HA, pBA002-3HA or pBA002-6Myc to generate HA-tagged and Myc-tagged constructs under the control of a 35S promoter. Point mutation in specific amino acids of AS2 were generated with Stratagene's QuikChange II Site Directed Mutagenesis Kit (Stratagene, USA) with primers listed in S1 Table.

pCAMBIA1300 vector was used to construct *AS2-EGFP*fusion gene expressed from a native *AS2* promoter (from -2.8kb to +2.0 kb, just before the initiation codon) to generate the plasmid 1300-AS2p(-2.8K):AS2-EGFP for stable transformation. NES from HIV Tat protein (LQLPPLERLTLD) and for NLS from SV40 virus (PKKKRKVKD) were used to make *AS2-NES* and *AS2-NLS*fusion genes. AS2 variants (*AS2-NES* and *AS2-NLS*) were replaced WT *AS2* in the plasmid of 1300-AS2p(-2.8K):AS2-EGFP and used for *AS2* native promoter driven AS2 variants expression vector. BV1 of CaLCuV was cloned into the pBA002-CFP to form pBA002-BV1-CFP.

### GFP imaging, antibodies and protein gel blot analysis

GFP imaging was as described [23]. To prepare protein samples, a leaf section (50mg) was ground in liquid N2 and extracted with 200 μl of 8M urea. The extract was mixed with 2 x SDS loading buffer and boiled for 10 min. Ten microliters of protein extract was separated by SDS-PAGE. Antibodies against GFP and protein gel blot analysis were as described in [23]. Transient silencing suppressor activity assays in *N*.*benthamiana* was performed as described [23]. For co-infiltrations, the OD_600_ of *GFP*-carrying agrobacterial strain was 0.8 and that of *AS2* was 1.2.

### RNA/DNA gel blot analysis

Total RNA was extracted from 12-day-old seedlings using Trizol reagent (Invitrogen) according to the manufacturer’s instructions. Fifteen μg total RNA were fractionated on a 1.2% (w/v) agarose gel and then transferred to a Hybond-XL membrane (GE Biosciences). Hybridization was performed overnight at 65°C in hybridization buffer (0.3 M sodium phosphate at pH 7.0, 10 mM EDTA, 5% SDS, 10% dextran sulfate, 0.15 mg/mL salmon sperm DNA), and signals were detected by autoradiography. For small RNA analysis, 15 μg of total RNA were fractionated on a 15% polyacrylamide gel containing 8 M urea and then transferred to a Hybond-N^+^ membrane (GE Biosciences). DNA oligonucleotides were end-labeled with [γ-^32^P] ATP using T4 polynucleotide kinase (New England Biolabs). Hybridization was performed overnight at 42°C using the ULTRAHyb-Oligo hybridization buffer (Ambion) and signals were detected by autoradiography. Southern blot was performed as described previously [26]. Membrane was hybridized with a probe encoding the AC1 (Rep) of CaLCuV DNA-A. The probe was DIG-dUTP-labeled by PCR using a PCR DIG probe synthesis kit (Roche Shanghai, China) and hybridization signals were detected by autoradiography.

### RNA extraction and quantitative real-time PCR (qRT-PCR) analysis

RNAs were isolated from leaf and stem samples using Qiagen RNeasy Plant mini kits (Qiagen) with on-column DNase treatment. Plant RNA purification reagent (Invitrogen) was also used for total RNA extraction followed with DNase treatment [27]. RNA concentration was measured by Nanodrop (Thermo, USA). M-MLV reverse transcriptase (Promega, USA) was used for reverse transcription reactions. Real-time PCR was performed with Power SYBR Green PCR Master (Applied Biosystems, USA) and run in ABI7900HT. All samples were run in triplicates and data was analyzed with RQ manager at a pre-set Ct value (Applied Biosystems, USA). PCR primers were listed in S1 Table. Cordycepin treatments and mRNA stability analysis were performed as described before [7]. Twelve-day-old seedlings were incubated in MS medium with cordycepin (3′-deoxyadenosine; Sigma-Aldrich) and DMSO treatment was used as a mock control. Data derived from the mock control was used for normalization for mRNA stability assays. Total RNA was extracted from samples harvested at various time points using Plant RNA extraction reagent (Invitrogen). *Actin2* was used as an internal mRNA control.

### *In vitro* pull-down assay

cDNAs encoding full-length CaLCuV BV1, AS1, AS2, DCP1, DCP2, DCP5 and VCS-C terminal were amplified by PCR using Phusion High-Fidelity DNA Polymerase (FINNZYMES) and subcloned to generate GST fusion, MBP fusion and 6His, 6His-SUMO fusion constructs. The yeast SUMO protein tag was used to improve AS2 protein solubility. All constructs were transformed into *E*. *coli* BL21(DE3) cells and cultured at 37°C. After the OD_600_ had reached ∼0.6, isopropyl β-D-thiogalactopyranoside was added to a final concentration of 0.4 mM and the culture incubated overnight at 20°C. Bacterial cells were collected by centrifugation and suspended in a lysis buffer containing proteinase inhibitor cocktail (Roche). After French press treatment, recombinant proteins were purified with specific affinity columns and AKTA FPLC system (GE Biosciences) followed by size-exclusion columns. The eluted proteins were concentrated by Ultracel YM-30 (Millipore). In vitro pull-down assays were performed with 2 μg of GST/MBP/His/His-SUMO fusion proteins. Proteins were incubated in a binding buffer (50 mM Tris-HCl at pH 7.5, 100 mM NaCl, 0.25% Triton X-100, 35 mM β-mercaptoethanol) for 2 h at 4°C, and 30 μl of glutathione sepharose 4B (GE Biosciences) were added and the mix incubated for overnight. After washing with binding buffer for 6 times, pulled-down proteins were separated on 12% SDS–polyacrylamide gel and detected by Western blotting using anti-His, anti-GST or anti-MBP antibody.

### Immunoprecipitation

About 3 g of 14-day old *Arabidopsis* seedlings expressing*35S*:*BV1-ECFP* or *AS2p(-2*.*8K)*:*AS2-EGFP* was used for ChIP assays. Seedlings were incubated with 50 μM MG132 (Calbiochem) for 12 hr before harvesting. Proteins were extracted in extraction buffer (50 mM Tris-HCl at pH 7.5, 150 mM NaCl, 2 mM MgCl_2_, 1 mM DTT, 20% glycerol, 0.5% nonident P-40) containing protease inhibitor cocktail (Roche) and protease inhibitor mixture (Sigma). Cell debris was pelleted by centrifugation at 14,000*g* for 30 min. The supernatant was incubated with GFP-agrose beads (GFPtrack) overnight at 4°C. Beads collected by centrifugation were washed 6 times with washing buffer (50 mM Tris-HCl at pH 7.5, 150 mM NaCl, 2 mM MgCl_2_, 1 mM DTT, 10% glycerol, 0.5% nonident P-40). Proteins were eluted by 50 μL of 2.5× sample buffer and analyzed by Western blotting using anti-DCP2 rabbit antibody [7].

### Chromatin immunoprecipitation (ChIP) assay

About 2 g of 14-day old *Arabidopsis* seedlings expressing *35S*:*BV1-ECFP* was used. Seedlings treated with 50 μM MG132 overnight were used for chromatin preparation and immunoprecipitation. Immunoprecipitation was performed by adding GFP-agrose beads (GFPtrack). After washing, immune complexes were eluted from protein A beads and reverse cross-linked by incubation for at least 6 h at 65°C. Samples were treated with proteinase K for 1 h at 65°C. DNA was extracted in a final volume of 80 μL using the QIAquick PCR purification kit (Qiagen). ChIP assay was repeated with 3 biological replicates. One microliter of DNA was used for each real-time quantitative PCR with SYBR Premix Ex Taq (Applied Biosystems, USA) in the ABI7900 real-time system (Applied Biosystems, USA). Each sample was assayed in triplicate by PCR. Error bars in each graph indicate standard deviation (SD) of three biological replicates. We used *ACTIN2* as an internal control. Primers used for ChIP assays are listed in S1 Table

### Bimolecular fluorescence complementation (BiFC)

BiFC was performed using vectors and methods described in [25]. Full length cDNAs encoding BV1 (CaLCuV), AS1, AS2, DCP1, DCP2, DCP5 and VCS-C terminal were cloned into corresponding restriction enzyme sites of BiFC vectors. The resulting cassettes including fusion genes and constitutive promoters were cloned into pGreen binary vector HY105 and transformed into *Agrobacterium*. For BiFC experiments, leaves of 3-week-old *N*.*benthamiana* plants were co-infiltrated with *Agrobacterium* as previously described. A nuclear protein Aux/IAA from *Jatropha curcas* was used as a nuclear marker [28]. IAA-ECFP was produced by PCR cloning into the pBA002-ECFP vector. The agrobacterium strain carrying IAA-ECFP was co-inflitrated with strains carrying BiFC vectors into *N*.*benthamiana* leaf cells. Images were taken from cells 48 hours post-infiltration using a confocal laser scanning microscope.

### *In vitro* decapping assay

RNAs (121nt-G16) containing a 16-guanine track at the 3′ end were transcribed with the MAXIscript T7 kit (Ambion) from a DNA fragment of *At2g38280* corresponding to the 5′ untranslated region (123 nucleotides) of the transcribed mRNA. To generate cap-labeled RNAs, the ScriptCap m7G Capping system (Epicentre) was sequentially used. Decapping assays were performed at 37°C for 30 min with cap-labeled RNA and the indicated amounts of purified proteins. Reaction products were resolved by thin layer chromatography as described by Xu J. et al. [7]. The reactions were performed in *in vitro* decapping assay buffer (10 mM Tris-Cl, pH 7.5, 100 mMKAc, 2 mM MgAc_2_, 0.5 mM MnCl_2_, 2 mM DTT, 0.1 mM spermine, and 25 μg/mL yeast tRNA). TLS PEI cellulose F plates (Merck) were used to resolve products of decapping assays.

## Results

### Shuttle protein BV1 of CaLCuV activates *AS2* expression in *Arabidopsis*

**Fig 1:**
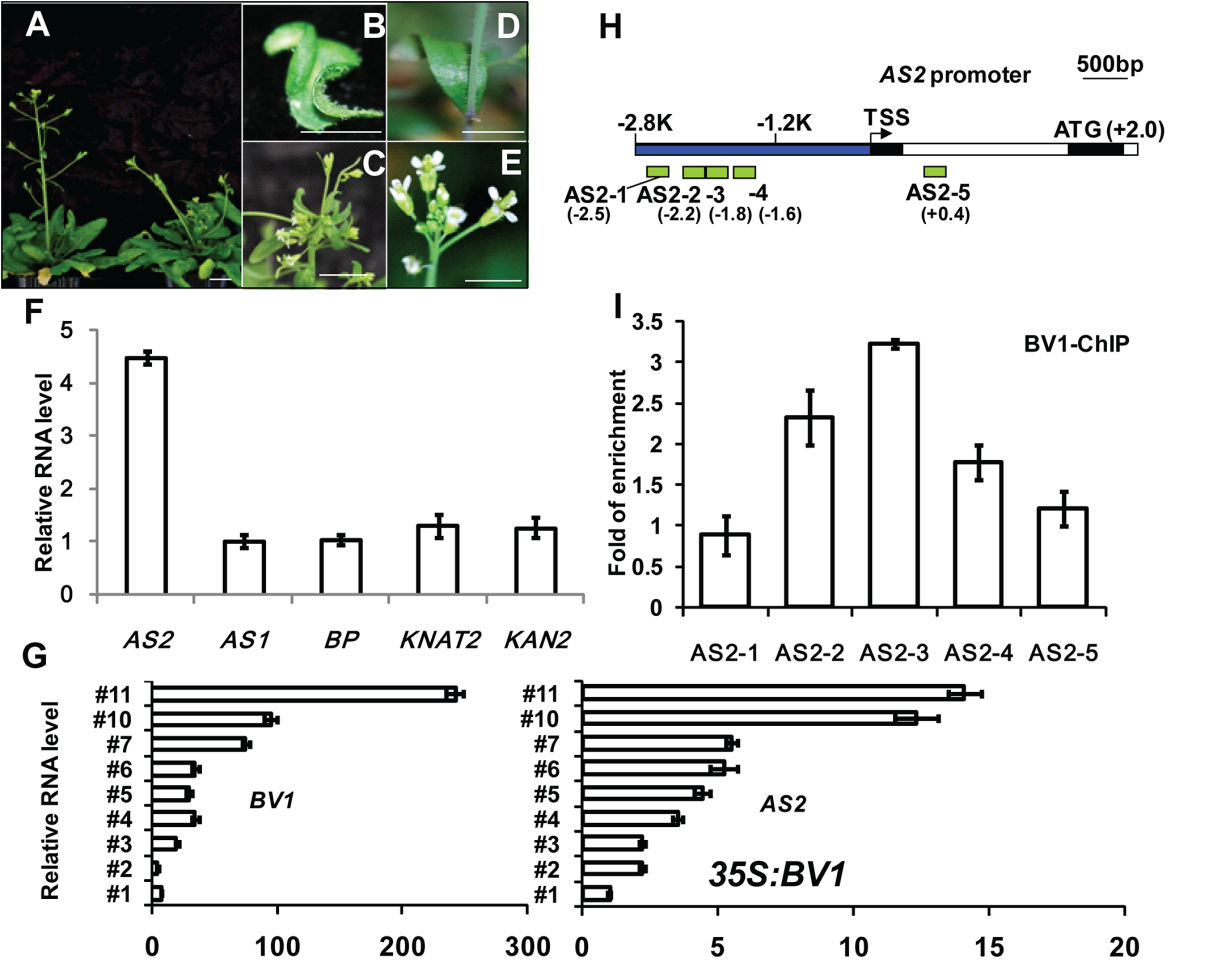
BV1 of CaLCuV activates *AS2* expression. (A-C) Arabidopsis plants infected with CaLCuV with BV1. (A) Left, a mock control *Arabidopsis* plant. Bar = 10 mm. Right, an *Arabidopsis* plant infected by CaLCuV showing typical symptoms. (D-E) *Arabidopsis* plants infected with mock (*BV1*-deficient CaLCuV). Bar = 10 mm. (F) Relative expression levels of *AS2*-related genes in CaLCuV infected plants compared to mock-infected plants. Values are mean ± SD (n = 3 biological replicates). (G) Relative expression levels of *AS2* and *BV1* in several L1 lines with different *35S*:*BV1* transgene expression levels (S1 Fig). Note the correlation in expression levels between *AS2* and *BV1*.Values are mean ± SD of three technical replicates. (H) Schematic diagram of the *AS2* genomic locus. The bent arrow indicates the transcription start site (TSS). The promoter region 5′ of TSS is shown in blue. Exons and introns are represented by black and white boxes, respectively. ATG indicates the translation start site. The hatched boxes represent the various DNA fragments amplified in ChIP assays. The end positions of each fragment (Kb) relative to the TSS are: AS2-1 (-2.6 to -2.5), AS2-2 (-2.3 to -2.2), AS2-3 (-1.9 to -1.8), AS2-4 (-1.7 to -1.6) and AS2-5 (+0.3 to +0.4). (I) ChIP analysis of BV1 binding to the *AS2* promoter. *35S*:*BV1-ECFP* seedlings were treated with MG-132 for 12 hr before being harvested for ChIP assays. Values are mean ± SD of three technical replicates. The fold enrichment was expressed relative to *AtACTIN2* which was used as an internal control. Amplification levels obtained with the individual primer pair served as background signal of BV1-ECFP binding. The experiment was repeated twice with similar results.

We infected *Arabidopsis* WT (Col-0) plants with *Cabbage leaf curl Virus* (CaLCuV), a model for bipartite geminivirus, and examined the symptoms of infected plants. CaLCuV infects *Arabidopsis* leaf tissues with high efficiency, inducing symptoms of severe stunting and leaf epinasty, suggesting inference with leaf development (Fig 1A, 1B and 1C, control plant shown in Fig 1D and 1E). To explore the molecular basis of the developmental abnormality, we determined transcript levels of a number of genes implicated in leaf development. Of the 5 genes tested only *AS2* transcript levels were obviously induced by more than 4 fold in infected plants (Fig 1F). By contrast, there was no *AS1* expression changes in the infected plants, although *AS1* shares overlapping function with *AS2* in leaf development. These results suggest that *AS2* may play a role in geminivirus pathogenesis and/or virulence, in addition to its known functions in leaf stem cell determination. Expression levels of *KNOTTED1*-like homeobox (*KNOX*) genes, *BREVIPEDICELLUS* (*BP*) and *KNAT2*, all of which are targets of AS2, were not visibly altered upon virus infection (Fig 1F).

The considerable increase in *AS2* transcript levels led us to hypothesize that AS2 may play a role in virus-host interaction. To see which viral component is responsible for the increased *AS2* expression, we generated transgenic *Arabidopsis* plants expressing several individual CaLCuV proteins. Preliminary analysis of transgenic plants identified the virulence factor BV1 as the candidate. Fig 1G and S1 Fig show a positive correlation between *BV1* expression and *AS2* expression in several independent transgenic lines. This result shows that BV1 alone may induce *AS2* expression, independent of any other viral factors.

The effect of BV1 on *AS2* expression may be direct or indirect. To test for possible direct binding of BV1 to the *AS2* genomic locus we performed Chromatin Immunoprecipitation (ChIP) assays using transgenic *35S*:*BV1-CFP* seedlings. Fig 1H and 1I show the- 2.2 Kb to -1.6Kb (600-bp) of the *AS2* promoter was more efficiently precipitated by BV1-CFP demonstrating possible binding of BV1 to this region of the *AS2* promoter.

To ascertain the importance of this *AS2* genomic region in BV1 interaction we constructed an *AS promoter*:*GUS* reporter gene (*AS2p*:*GUS*). Owing to the long intron in the 5'UTR of *AS2* (Fig 1H) the reporter gene consisted of 4.8Kb *AS2* genomic sequences (from -2.8kb to +2.0 kb, just before the initiation codon) fused to a *GUS* coding sequence downstream (*AS2p*:*GUS*). We transformed *Arabidopsis* plants of different genotypes with the *AS2p*:*GUS* fusion gene. GUS activity was high in WT (Col-0) young seedlings (S2A Fig) but weaker in mature leaves and inflorescence stems confirming previous results [20]. Infection experiment verified the *AS2p*:*GUS* activity was virus-inducible in a BV1-dependent manner (compare right and left plants in S2C Fig). By contrast, *AS2p*:*GUS* activity was low in *as2-1* mutant (S2B Fig) suggesting positive auto-regulation by AS2 (Compare uninfected plants shown in S2A and S2B Fig, also infected plants shown in S2D and S2E Fig), in addition to its activation by BV1. BV1-dependent *AS2p*:*GUS* activity was much reduced when a 1.6 Kb sequence (-2.8 to -1.2Kb) of *AS2* 5′ upstream region was deleted from the promoter of the reporter gene (S2F Fig). This result is consistent with the chromatin immunoprecipitation data that BV1 associated with this 1.6 Kb region to activate *AS2* promoter activity.

**Fig 2:**
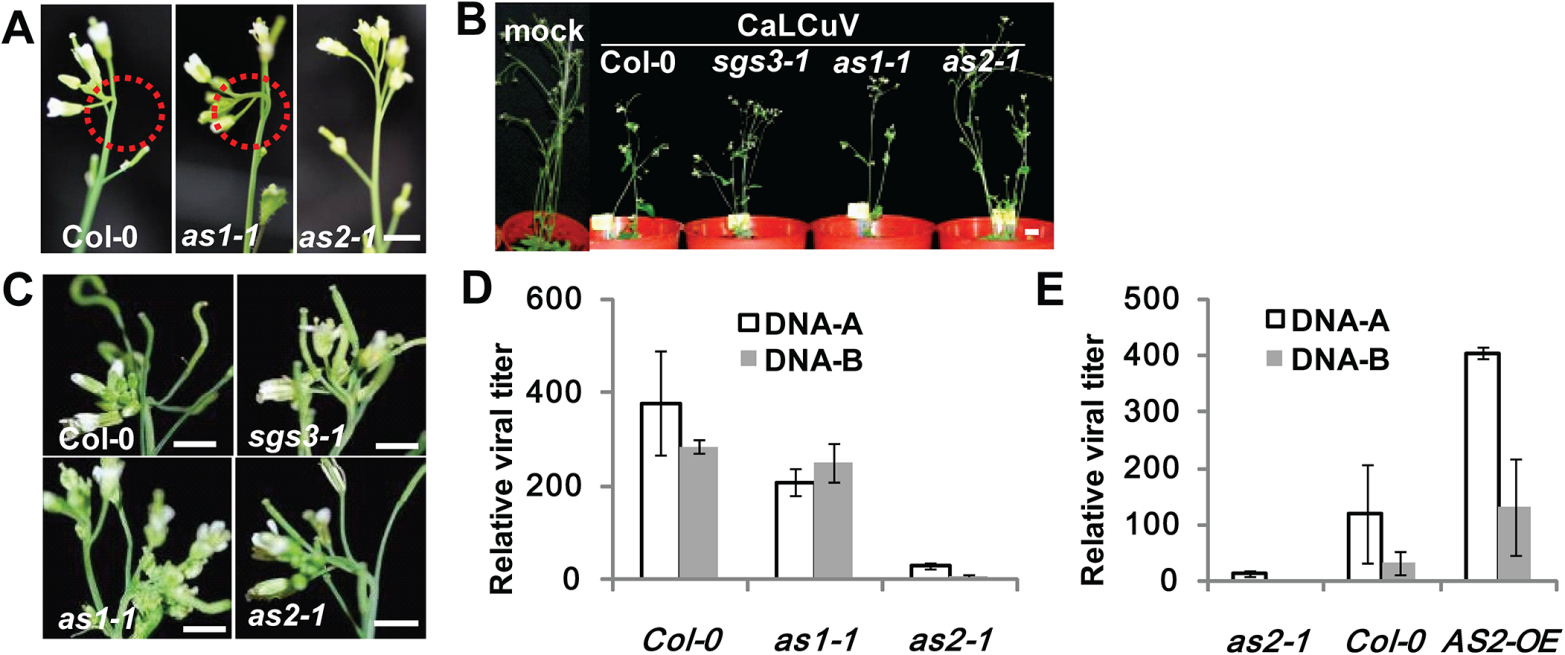
*as2* mutant is resistant to CaLCuV infection. (A) WT (Col-0), *as1-1* or *as2-1* plants infected with CaLCuV. Note typical viral symptoms of early-stage flowers (10 dpi) in WT and *as1-1* inflorescence. Bar = 10mm. (B) WT and mutant plants infected with CaLCuV. Pictures were taken at 28 dpi. Bar = 10mm. (C) Flowers of WT and mutant plants from (B) (28 dpi). Bar = 10mm. (D) Relative CaLCuV DNA levels (DNA-A and DNA-B) in infected (by particle bombardment) WT, *as1-1*, and *as2-1* plants (28 dpi). *Actin2* levels served as an internal plant genomic DNA control. Values are mean ± SD (n = 3 biological replicates). (E) Relative CaLCuV DNA levels (DNA-A and DNA-B) in infected (by particle bombardment) WT, *as2-1*, and *35S*:*AS2* overexpression (*35S*:*AS2*) plants (18 dpi). *AtActin2* levels served as an internal plant genomic DNA control. Values are mean ± SD (n = 3 biological replicates).

Two opposing hypothesis may be proposed to explain the activation of *AS2* by BV1. One possibility is that the host protein may be induced as part of the host defense system to counter and block virus infection. The other possibility is that the AS2 induction may underpin a subversive mechanism used by the virus to compromise host defense. To discriminate between these two possibilities, we infected *Arabidopsis* WT and mutant plants of different genotypes: *as2-1*, *as1-1*, two related leaf developmental mutants (phenotypes shown in S3A Fig) [19] and *sgs3-1*, an enhanced susceptibility mutant [29]. Fig 2 shows viral infection symptoms were pronounced on WT plants as well as plants of *as1-1* and *sgs3-1*. Moreover, consistent with published results [29] the viral symptomatic onset on *sgs3-1* plants was 2–3 days earlier compared with WT control and *as1-1*. There was no obvious difference in viral symptomatic onset between WT and *as1-1* plants. In contrast to *sgs3-1*, the onset of viral symptoms on *as2-1* was 2–3 days later than in WT and *as1-1* (Fig 2A, 12 dpi). At the late stage (28 dpi), there was no obvious symptomatic difference between WT and *sgs3-1*, whereas *as1-1* displayed a slightly weaker symptom (Fig 2B). Much milder symptoms including curling of leaves, flowers and siliques and dwarfing of plant stature were found with infected *as2-1* plants (Fig 2B and 2C). These results indicate AS2 deficiency attenuates virus replication.

To obtain quantitative data on viral titers we determined viral genomic DNA accumulation levels in CaLCuV-infected plants of different genotypes. Plants of *as2-1* were more resistant to the geminivirus as compared to *as1-1* and WT plants. The virus titer was much lower in systemic leaf of *as2-1* either by Southern blot or quantitative PCR (Q-PCR) analysis, suggesting AS2 interferes with virus replication (S3B Fig and Fig 2D). The results so far suggested that AS2 may function as a negative regulator of host defense mechanisms and its deficiency would lead to virus resistance. If this hypothesis was correct, then AS2 overexpression should produce the opposite results. Fig 2F and S4 Fig show that this was indeed the case both in *Arabidopsis* and *N*. *benthamiana*. *35S*:*AS2* over-expression *Arabidopsis* and *N*. *benthamiana* plants were much more sensitive to bipartite geminiviruses, CaLCuV and *Indian Cassava Mosaic virus* (ICMV) respectively, with high viral DNA-A and B levels.

### AS2 overexpression can block PTGS in *Arabidopsis* and *N*. *benthamiana*

**Fig 3:**
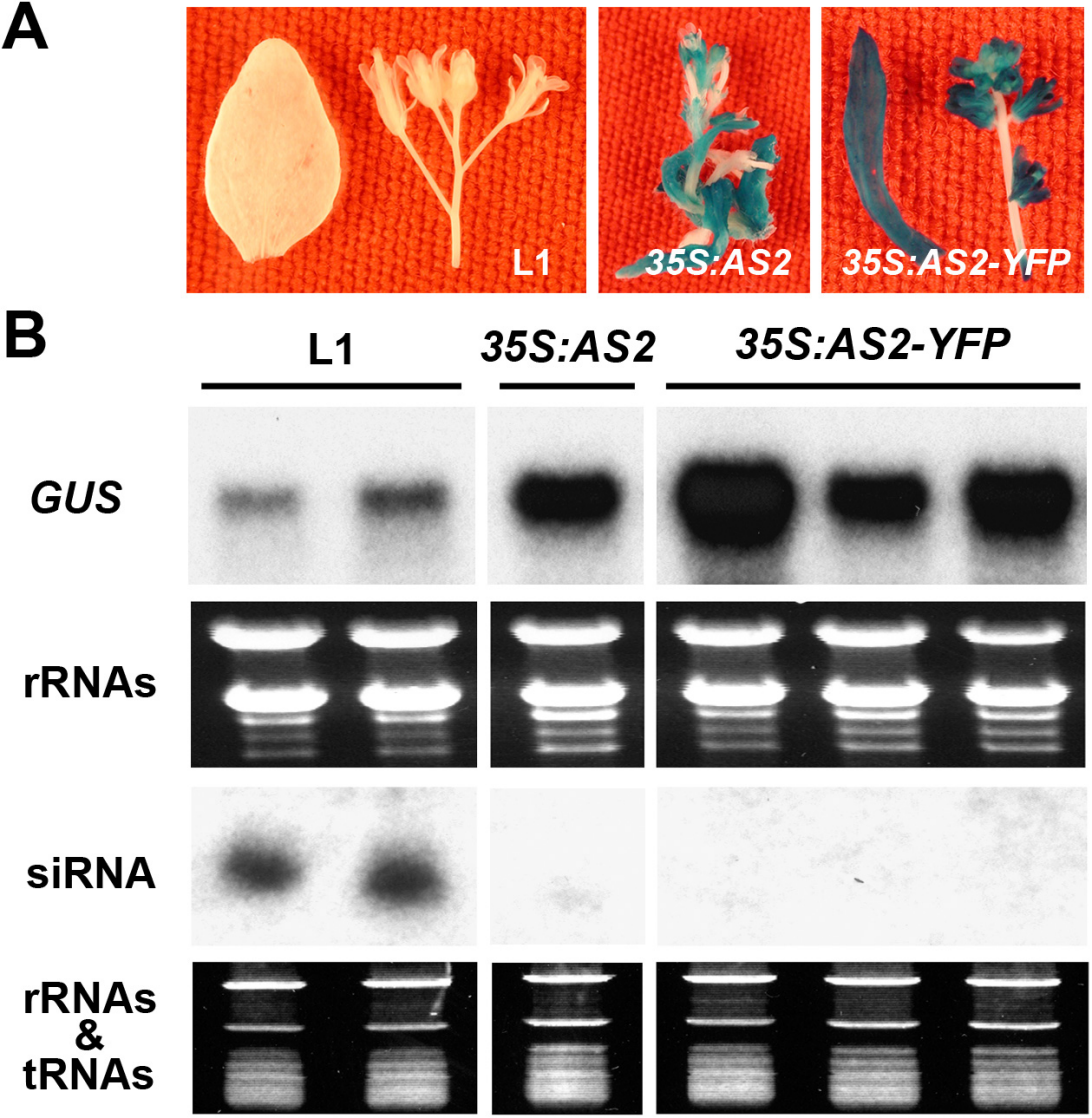
*AS2* overexpression blocks PTGS in *Arabidopsis*. (A) The *Arabidopsis* L1 line (Col-0 background) carries a *35S*:*GUS* that has been silenced by PTGS [24]. The L1 line was re-transformed with *35S*: *AS2* and *35S*:*AS2-YFP*. Note the re-activation of GUS expression by *35S*: *AS2* and *35S*:*AS2-YFP*. Bar = 10 mm. (B) *GUS* mRNA and *GUS-*specific siRNA analysis in L1, *35S*: *AS2*/L1 and *35S*:*AS2-YFP*/L1 lines.

To directly confirm AS2 suppressor activity, we expressed *35S*:*AS2* in the L1 line of *Arabidopsis thaliana* which harbors a silenced *GUS* gene owing to PTGS. Fig 3A shows that AS2 overepxression reactivated the silenced *35S*:*GUS* transgene in the L1line. In reactivated plants, the increased *GUS* mRNA levels were accompanied by decreased *GUS*-related siRNA levels as compared to the parental L1 line (Fig 3B). Similar results were obtained by over-expression of a *35S*:*AS2-YFP* fusion gene (Fig 3A and 3B). We also tested AS2 suppressor activity by transient expression in *N*. *benthamiana* using the strong viral suppressor P19 of Tomato bushy stunt virus as a control. S5 Fig confirms the suppressor activity of AS2 and less small interfering RNA (siRNA) accumulation by co-expression of AS2 also shows that its activity was weaker compared to P19. Together, these results indicate that AS2 can function as an endogenous PTGS suppressor in two different assay systems.

### Cellular localization of AS2

**Fig 4:**
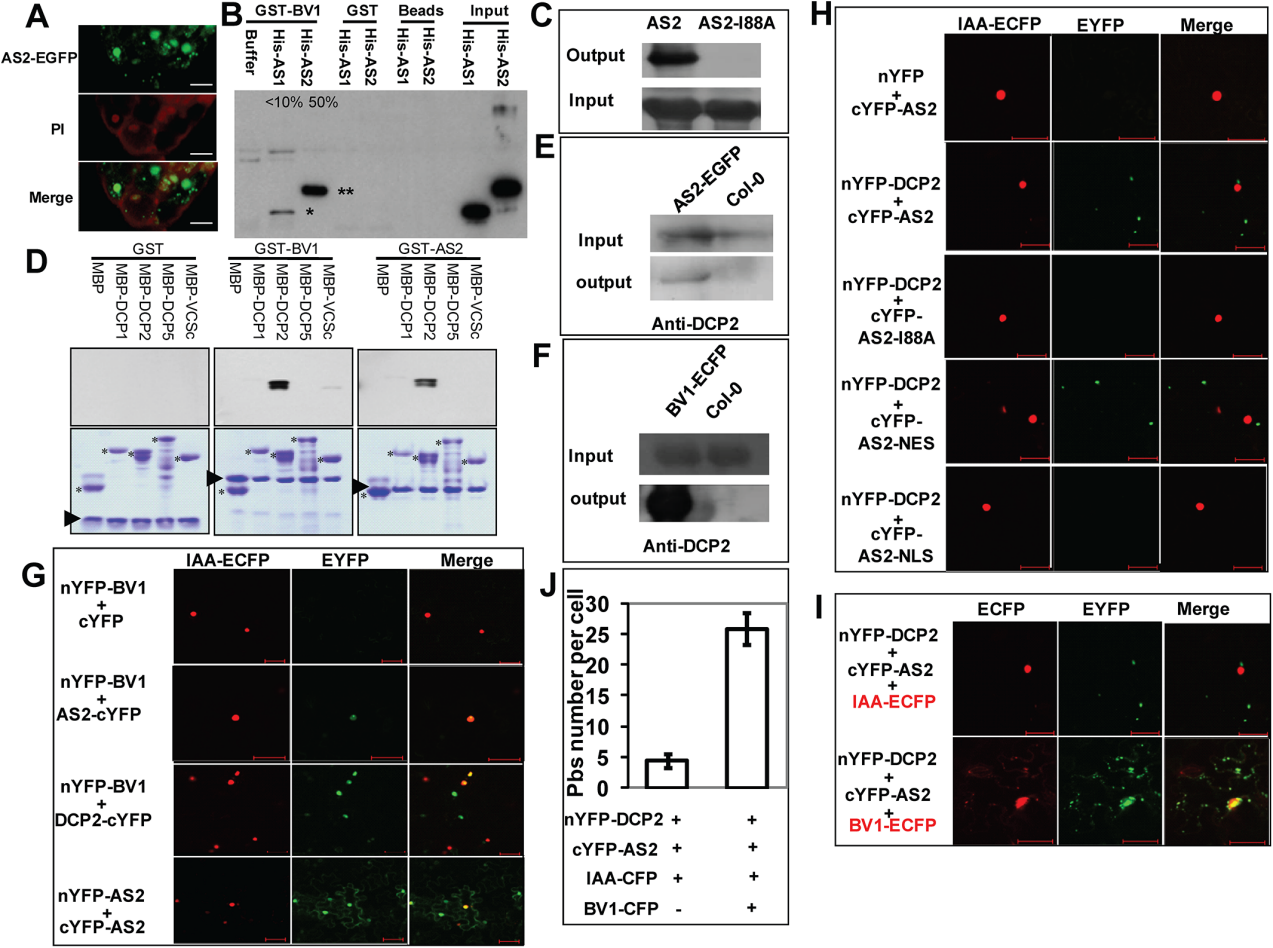
BV1 interacts with AS2 and DCP2 (A) Sub-cellular localization of AS2-EGFP fusion protein expressed from a native *AS2* promoter in transgenic *Arabidopsis* root. Propidiumiodide (PI) was used to stain the nucleus and plant cell wall. Note cytoplasmic AS2 bodies. Bar: 10μm. (B) *In vitro* interaction of BV1 with AS1 and AS2. Around 50% of the input AS2 protein can be pulled down by GST-BV1, whereas less than 10% of the AS1 protein can be pulled down by GST-BV1. (C) *In vitro* assay of self-association of WT AS2 and its I88A mutant. Equal input amounts of His-AS2 and His-AS2-I88A were used for GST-AS2 pull down assays. (D) *In vitro* interaction of BV1 or AS2 with various protein subunits of the P-body. Arrowheads and asterisks indicate the bait proteins and the prey proteins, respectively, in each experiment. (E) *In vivo* interaction between AS2 and DCP2. Nuclear extracts from *AS2p*:*AS2-EGFP* plants were immunoprecipitated by GFP-agrose beads (GFPtrack). The immunoprecipitate was detected by anti-DCP2 antibody. (F) *In vivo* interaction between BV1 and DCP2. Nuclear extracts from *35S*:*BV1-ECFP* plants was immunoprecipitated by GFP-agrose beads (GFPtrack). The immunoprecipitate was detected by anti-DCP2 antibody. (G) BiFC assay. cDNAs encoding proteins fused to the N- or the C-terminal portion of YFP were constructed. Aux/IAA-ECFP (IAA-ECFP) was used as a nuclear marker. The co-expressed proteins are indicated. Bar = 50μm. (H) BiFC assay of *in vivo* interaction of DCP2 with AS2, AS2-I88A, AS2-NES and AS2-NLS. Aux/IAA-ECFP (IAA-ECFP) was used as a nuclear marker. The co-expressed proteins are indicated above the photographs. Bar = 50μm. (I) BV1 expression triggers nuclear exit of AS2-DCP2 to the cytosol. Aux/IAA-ECFP (IAA-ECFP) was used as a nuclear marker and also a negative control for BV1-ECFP. The co-expressed proteins are indicated above the photographs. Bar = 50μm. (J) BV1 expression increases the number of P body marked by DCP2-AS2 complex. Twenty cells were examined in this experiment.

**Fig 5:**
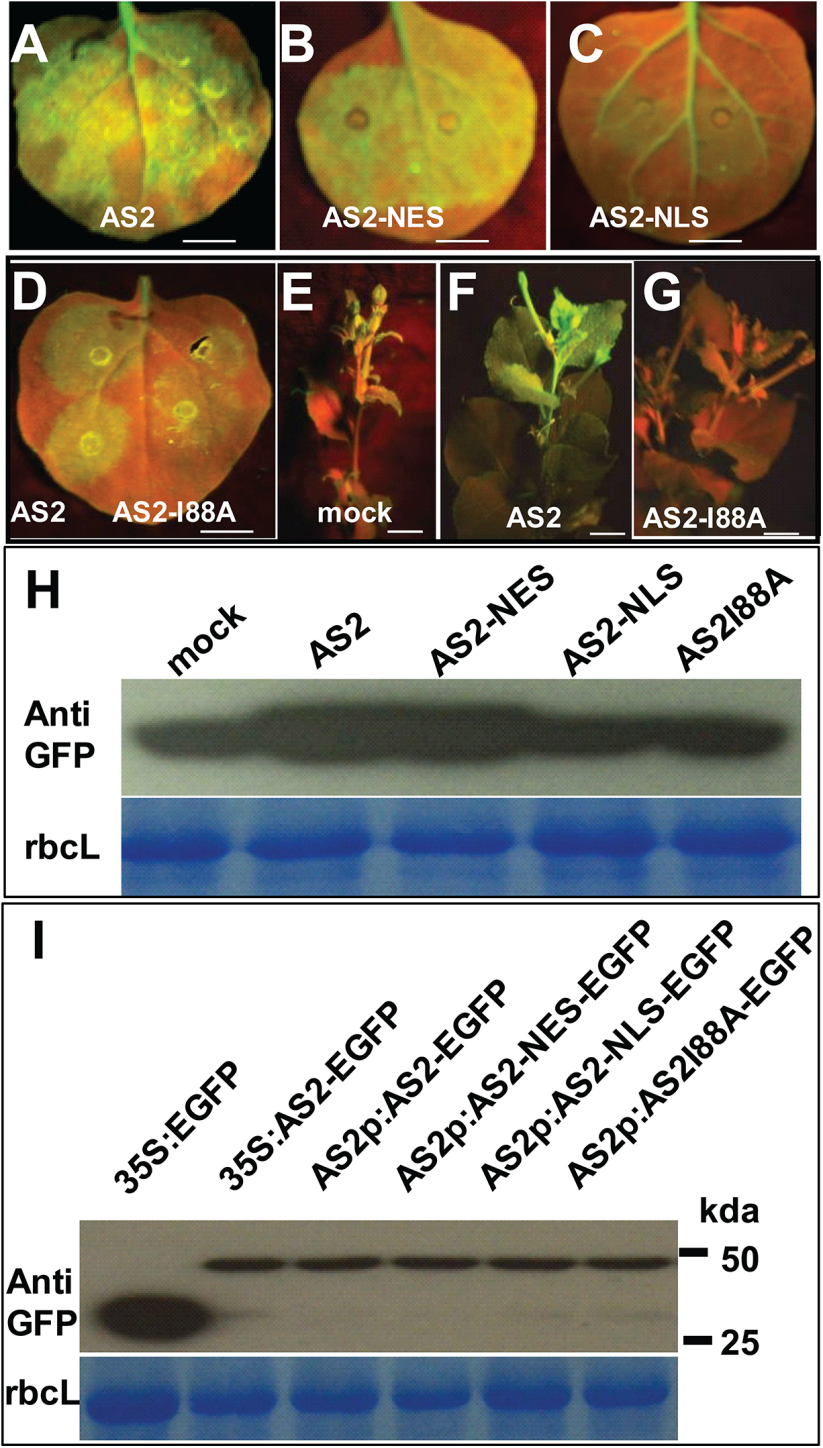
Cytosolic AS2 is a functional PTGS suppressor. (A-G) Suppressor activity of AS2, AS2-NES, AS2-NLS and AS2 I88A mutant. *N*. *benthamiana* (line 16c) leaves were co-infiltrated with *35S*:*GFP* and *35S*:*AS2* or 35S:*AS2-NES* or *35S*:*AS2-NLS* or *35S*:*AS2I88A* (local silencing, A-D). E-G show systemic silencing. AS2 can interfere with systemic silencing induced by *35S*:*GFP* (compare 5F of *35S*:*AS2* co-infiltrated with 5E of mock co-infiltrated with *35S*:*GFP* and 5G with AS2 I88A mutant). Bar = 10 mm. Bar = 10 mm. (H) Western blot analysis showing GFP protein levels in *N*. *benthamiana* leaf (line 16c) infiltrated with *35S*:*GFP* along with *35S*:*AS2*, *35S*:*AS2-NES*, *35S*:*AS2-NLS*, *35S*:*AS2I88A*, or mock treatment (upper panel). Coomassie blue-staining of the large subunit of ribulose 1,5-bisphosphate carboxylase/oxygenase (rbcL) is shown as a loading control (lower panel). (I) Western blot analysis showing AS2 and its variants protein levels in *N*. *benthamiana* leaf. Coomassie blue-staining of the large subunit of ribulose 1,5-bisphosphate carboxylase/oxygenase (rbcL) is shown as a loading control (lower panel).

AS2 was first characterized as a transcription factor which interacts with AS1 to regulate a number of downstream genes involved in leaf development [19]. This observation implies that the AS2 protein should be nuclear localized in order to execute its transcriptional function. On the other hand, our results above indicate that AS2 can function as a suppressor of PTGS, which is known to occur in the cytosol. This discrepancy can be resolved if AS2 can shuttle between the two cellular compartments to execute different functions. In fact, when expressed from its native promoter the AS2 protein was found in both the nuclear and the cytosolic compartment in transgenic *Arabidopsis* roots (Fig 4A and S6 Fig). Nuclear localization of AS2 has been previously reported [20]. Similar dual subcellular localization of the AS2 protein was seen by transient expression in tobacco leaves (S7 Fig) To identify the cellular compartment in which AS2 carries out its suppressor activity, we fused AS2 with either a Nuclear Localization Signal (NLS, Simian vacuolating virus 40, SV40) or a Nuclear Exporting Signal (NES, human immunodeficiency virus, HIV). The two AS2 localization mutants were tested for their suppressor activity. Fusion of NES did not interfere with AS2 suppressor activity (Compare Fig 5A and 5B, also 5F) but NLS fusion greatly attenuated AS2 suppressor activity (Compare Fig 5A and 5C, also 5H). Furthermore, protein level analysis indicated that AS2 and AS2 variants have comparable stability (Fig 5I). These results provide evidence that cytosolic localization of AS2 is required for its PTGS suppressor activity.

Furthermore, a mutation that converts a conserved amino acid Ile 88 to Ala blocked local and systemic silencing suppressor activity (S8 Fig and Fig 5D to 5I).

### AS2 and BV1 interact with decapping proteins

Because BV1 is a nuclear-cytosol shuttle protein we asked whether BV1 was able to bind AS2 in addition to activating its encoding gene. Fig 4B shows BV1 interacted strongly with AS2 but much less with AS1 in pull-down assays *in vitro*. AS2 was capable of self-interaction forming dimers or even higher-order protein complexes. This self-association property was compromised by the I88A mutation (Fig 4C).

To determine the mechanism of AS2 action in PTGS we screened for possible protein-protein interaction with proteins in the PTGS pathway and also proteins involved in RNA quality control machinery. *Arabidopsis* DCP2 has been recently reported as a negative regulator for PTGS [10]. This observation prompted us to examine whether BV1 and AS2 may interact with protein subunits of the decapping complex [7]. Using either BV1 or AS2 as a bait we found that AS2 strongly interacted with DCP2 and weakly with DCP1 in vitro (Fig 4D). Moreover BV1 also weakly interacted with the COOH- terminal fragment of VARICOSE (VCS). The strong interaction between either BV1 or AS2 with DCP2 was further confirmed by co-immunoprecipitation experiments (Fig 4E and 4F).

To confirm the observed protein-protein interactions *in vivo*, we performed Bimolecular Fluorescence Complementation (BiFC) using yellow fluorescent protein (YFP). We generated constructs to express AS2, BV1 or DCP2 fused at their N- or C- termini with the N- or C- terminal portions of YFP (nYFP and cYFP). Expression constructs were introduced into *N*. *benthamiana* leaf cells by agroinfiltration, and those with complementary YFP fusions (i.e. nYFP + cYFP) were analyzed by confocal microscopy 48 hours post-infiltration.

Test proteins were examined in all possible combinations. No signal was detected in control experiments in which only one fusion protein was expressed. Co-expression of the paired BV1/AS2 and BV1/DCP2 proteins resulted in YFP fluorescence in nuclei foci indicating complex accumulation in these subcellular locations. AS2/AS2 paired protein signals were found in both nuclear and cytosolic regions (Fig 4G). By contrast, the fluorescence signal of AS2/DCP2 pair was exclusively localized in cytosolic speckles, corresponding most likely to P bodies. No signal was found in the nucleus (Fig 4H).

Next, we examined the effect of the I88A mutation on interaction with DCP2. BiFC analysis showed that amino acid I88 was important for AS2/DCP2 association in *N*. *benthamiana* cells (Fig 4H).

Since BV1 is a shuttling protein and interacts with AS2 it is possible that this viral protein may shuttle nuclear AS2 to the cytosol to function as a silencing suppressor. To this end, we co-expressed BV1-CFP in the presence of the AS2/DCP2 BiFC combination. Many cytosol P-bodies like structures with CFP/YFP co-localization were observed (Fig 4I) and the results were confirmed by statistical analysis (Fig 4J). Taken together, our results suggest that the formation of multiple protein bodies (BV1-AS2-DCP2) may play important roles in BV1 and AS2 silencing suppressor activity.

### AS2 is able to promote decapping enzyme activity of DCP2

**Fig 6:**
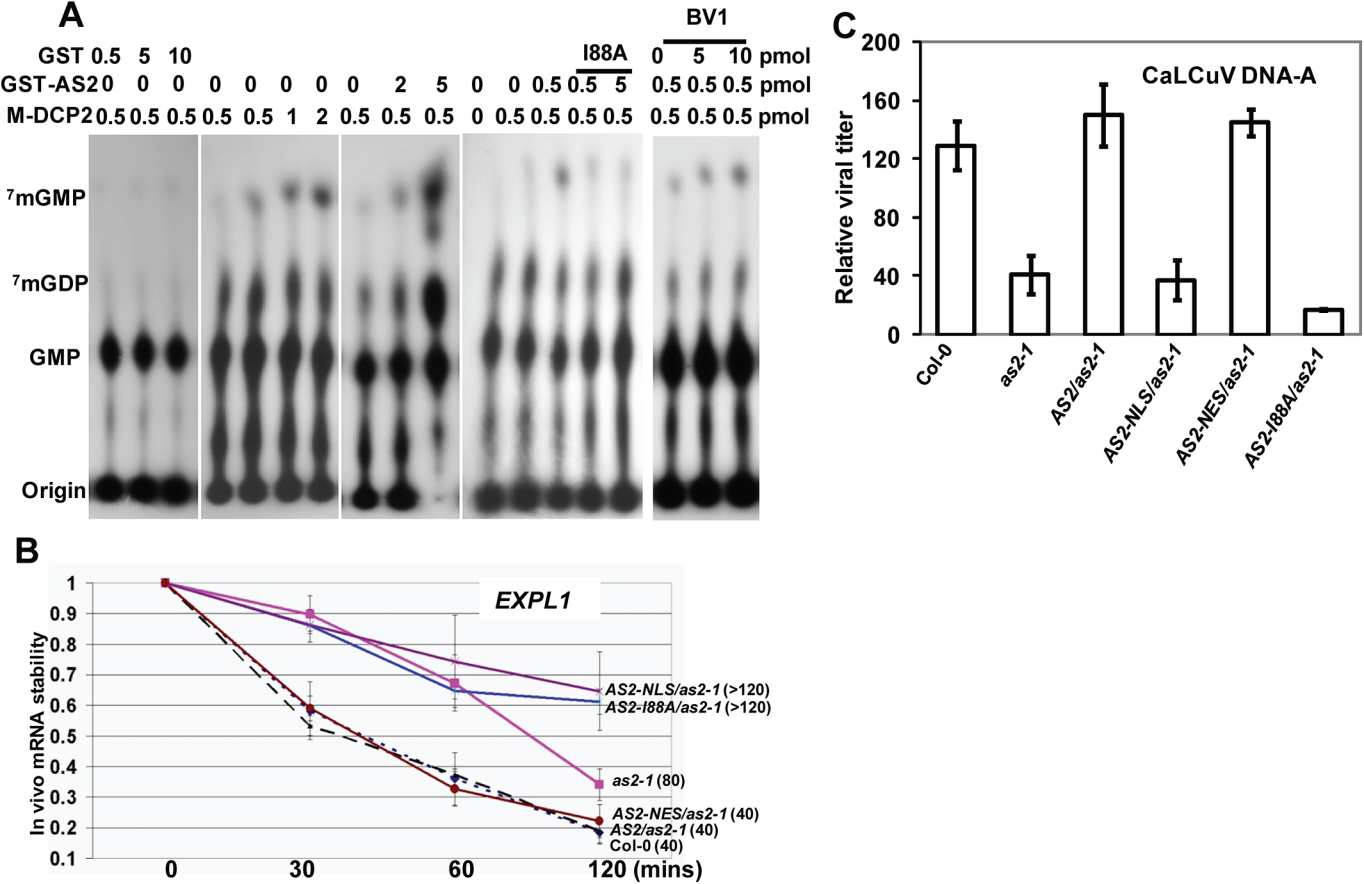
Decapping machinery antagonizes PTGS. (A) AS2 promotes DCP2 decapping activity in vitro. Addition of BV1 can further increase the ability of AS2 to enhance the decapping activity of DCP2. (B) *In vivo AtEXPL1* mRNA stability of *as2-1* mutant and AS2 complementation lines after cordycepin treatment. In WT (Col-0) plants, the half-life(½t) for *AtEXPL1*mRNA was 40 min. The level of *AtEXPL1* mRNA in each time point was normalized against that of solvent (DMSO) treatment. *Actin2* was used as an internal control. Values are mean ± SD (n = 3 biological replicates). (C) Relative virus titer of AS2 complementation lines infected with CaLCuV. Values are mean ± SD (n = 3 biological replicates).

Among the various P body components only DCP2 has been shown to possess decapping activity [13]. Therefore, we examined the effect of AS2 on DCP2 decapping activity in vitro. Fig 6A shows that DCP2 decapping activity was stimulated by the addition of AS2 and this stimulating effect was abolished by the I88A mutation. There was no obvious difference in the stimulating effect on DCP2 decapping activity between WT AS2 and its localization mutant derivatives (AS2-NLS and AS2-NES) (S9 Fig), which still retained the ability to physically interact with DCP2. Taken together, these results indicate that the cytoplasmic localization of AS2 *in vivo* is indispensable for its silencing suppressor activity. BV1 also showed a moderate stimulating effect on DCP2 decapping activity *in vitro* (Fig 6A).

We performed qRT-PCR analysis to test whether mRNA turnover was altered in *as2-1* and in plants expressing AS2 and derivatives. To this end, we used the native *AS2* promoter to express coding sequences for AS2-EGFP, AS2-I88A-EGFP, AS2-NLS-EGFP and AS2-NES-EGFP in the *as2-1* mutant background and the resulting transgenic plants were tested for mRNA stability *in vivo*. *Expansin-Like1* (*EXPL1*) transcript levels were 4-fold in *as2-1* mutant than that of in the WT control (S10 Fig). Moreover, Fig 6B shows the estimated half-life of *EXPL1* transcripts was about 80 min in *as2-1*, >120 min in *AS2-NLS-EGFP* and in *AS2-I88A-EGFP* plants. These values were at least two times higher than the half-life of 40 min found in WT control samples (Fig 6B). We also observed that the half-life of *EXPL1* transcripts was about 40 min for *AS2-EGFP* and *AS2-NES-EGFP* plants. These *in vivo* results support the notion that AS2 increases the decapping capacity of DCP2 in the cytosol to accelerate mRNA decay *in vivo*.

### Virus resistance of AS2 cellular localization mutants

If the ability of AS2 to suppress PTGS is executed mainly in the cytosol then an AS2 mutant that is exclusively localized in the nucleus should be ineffective whereas a mutant that is localized to the cytosol should remain active. To test this hypothesis we used the transgenic lines generated above with appended NLS and NES sequences to direct the AS2 protein to specific subcellular localization. Fig 6C shows that whereas *as2-1* mutant was resistant to CaLCuV the complemented line (AS2/*as2-1)* was as sensitive to the virus as WT. Addition of the NLS sequence to retain AS2 protein in the nucleus resulted in transgenic plants that were as sensitive as *as2-1* mutant plants indicating a lack of complementation for PTGS suppression. On the other hand, addition of NES sequence to AS2 produced the same result as WT AS2 indicating that PTGS function was mediated by cytosolic AS2. Quantitative analysis on virus titer in infected plants further confirmed these results (Fig 6C)

## Discussion

### Roles of RNA decapping in plant virus resistance

Host decapping system plays important roles in host/viral pathogen interaction in several systems but whether increased decapping activity is a host anti-viral defense or a viral strategy to weaken host defense depends on individual cases. For some animal and human viral pathogens, e.g. positive-stranded or negative-stranded RNA viruses, the mRNA decapping machinery is an important host immune system to counter virus infection [11]. On the other hand, mRNA decapping in cytoplasmic P-bodies is also essential for virus to complete their life cycle in animal or human cells, e.g. FHV [30] and therefore, in this case, increased decapping activity would presumably aid the pathogen. So far, the only viral encoded decapping enzyme, the vaccinia D10, is synthesized at a later stage of the virus life cycle, after viral DNA replication. D10 synthesis correlates well with the shutdown of host gene expression, and deletion of this gene has been shown to cause persistence of host and viral mRNAs in infected cells [31]. The vaccina D10 decapping enzyme may help restrict host antiviral responses by accelerating host mRNA degradation during poxvirus infection. The Kaposi's sarcoma-associated herpesvirus encodes a host shutoff factor SOX which commandeers cellular mRNA turnover pathways to destroy host mRNAs following digestion by cellular exonuclease Xrn1. This result suggests that Xrn1 is poised to deplete mRNAs undergoing translation in mammalian cells [32]. Based on these observations, a very likely scenario is that viral component(s) may hijack host cellular mRNA turnover machinery to efficiently destroy or enhance host mRNAs or aberrant viral RNAs transcribed from DNA or RNA viruses. Our data here presents a new mechanism by which plant pathogens mis-regulates host cellular mRNA turnover machinery to attenuate host anti-virus defense. This finding may aid in advancing knowledge on molecular mechanisms of host-virus interaction in animal pathogens. Meanwhile, DCP2 and decapping machinery may also contribute to innate immune response by a negative feedback mechanism to restore normal homeostasis following viral infection [33].

Compromising cytoplasmic or nuclear 5'-3' exoribonuclease function enhances transgene PTGS in *Arabidopsis*, suggesting that these pathways compete for similar RNA substrates. The *Arabidopsis* DCP2 is a negative regulator for RNA silencing in *Arabidopsis* [10] and *N*. *benthamiana* (this study, S5 Fig) indicating its inhibitory role on PTGS. Competition between single-stranded RNA substrates between RNA quality control and PTGS ensures appropriate partitioning of RNA substrates among these RNA degradation pathways [34]. Mutants of *UPF1*, a gene in Nonsense-Mediated Decay pathway, showed hypersensitivity to (+) RNA virus PVX infection, further highlighting the complexity of RNA substrates partitioning for virus immune response [35].

### AS2 is a new component of mRNA decapping machinery and functions as an endogenous PTGS suppressor

In higher plants, more recently also in mammals, PTGS has emerged as a vital antiviral resistance machinery that competes with the decapping system for RNA substrates. PTGS suppresses viral gene expression through the production of dsRNAs and siRNA-directed viral RNA cleavage, mainly in the cytoplasm. On the other hand, aberrant RNA substrates are depleted by decapping system inhibiting PTGS. This substrate competition implies that increased decapping activity would block PTGS and is expected to aid virus virulence.

AS2 has been well-characterized as a nuclear factor that transcriptionally regulates several downstream genes involved in leaf development. Here, we identified AS2 as a new regulatory component of *Arabidopsis* cytosolic P body, which activates DCP2 decapping activity *in vitro* (Fig 6A) and probably *in vivo* as reflected by the *EXPL1* mRNA stability *in planta* (Fig 6B). We provide evidence that AS2 also functions as an endogenous PTGS suppressor: 1) AS2 is able to restore expression of a silenced transgene (Fig 3); 2) *as2* mutant shows resistance to virus infection (Fig 2 and Fig 6); 3) *AS2* overexpression blocks PTGS and promotes virus infection (S5 Fig and S4 Fig); 4) Cytosolic but not nuclear AS2 inhibits PTGS and siRNA accumulation (Fig 5 and S5 Fig); 5) Nuclear-localized AS2 variant is not able to rescue the virus sensitivity phenotype of *as2* mutant (Fig 6C); 6) Nuclear-localized AS2 variant is not able to rescue the mRNA turnover defect of *as2* (Fig 6B) although this variant retains similar capacity as WT AS2 in promoting decapping activity in vitro (S9 Fig).

The biological activity of the AS2/DCP2 complex is further reflected in similar morphological phenotype of monogenic mutants. The venetion pattern of *as2-1* cotyledons was disrupted similar to that found in *dcp2-1* suggesting that the two genes, *AS2* and *DCP2*, operate in the same pathway. Furthermore, we found that the *AS2* mRNA itself is feed-back regulated by the decapping complex, since *AS2* transcript levels are elevated by approximately two-fold in decapping mutants [7]. This feed-back mechanism is also found in *dcp1*, *dcp2* and *vcs* mutants [7].

In addition to AS2 a few **e**ndogenous silencing suppressor for PTGS in plants have been previously identified, including the cytoplasmic exoribonucleases, XRN2, XRN3 and XRN4 and DCP2 [9,10,18,36]. Given the importance of the decapping system in inhibiting PTGS we would expect any potential cellular activators of DCP2 activity to work as endogenous suppressors of PTGS as well. Likely candidates include DCP1 and DCP5 [7,8].

### BV1 induces AS2 expression and translocates AS2 into cytosolic decapping body

The begomovirus DNA-B component encodes two proteins, the BV1 (nuclear shuttle protein, NSP) and the BC1 (movement protein, MP), both being required for systemic infection. BV1 which shuttles between the nucleus and the cytoplasm is believed to mediate the intracellular trafficking of viral DNA. BV1 also interacts with jasmonate singalling regulator MYC2 and plant immune receptor NIK1 to counter host resistance to pest and disease [15,25]. Here, we identified the geminivirus BV1 protein as a virulence factor that promotes mRNA decapping efficacy in P bodies so as to indirectly attenuate PTGS. BV1 is a nuclear-cytoplasmic shuttle protein and can bind single or double stranded DNA or RNA with no known sequence preference. The effects of BV1 on decapping activity are both direct and indirect. BV1 can induce expression of the nuclear gene *AS2* but the *AS2* induction by BV1 or virus does not lead to increased expression levels of downstream genes, e.g. *KNAT* etc (Fig 1F). This observation suggests that most of the induced and newly-synthesized AS2 are escorted by BV1 into the cytosol to enhance degradation of aberrant RNAs, therefore reducing the RNA substrate for siRNA biogenesis. In addition to this indirect effect, BV1 also weakly activates the activity of RNA decapping, through which some plant transcripts involved in antiviral immunity may be reduced faster. A recent report showed that BV1 may interfere with host antiviral immunity by translation suppression of host proteins [37]. Meanwhile, based on the fact that BV1 mainly interacts with AS2 in the nucleus (Fig 4G), it is also possible that DCP2 drags part of BV1 out of the nucleus

Beside of its involvement in activating decapping we cannot exclude other functions of BV1 on begomovirus pathogenesis, e.g. direct siRNA sequestration, which is dependent or independent of AS2 induction of decapping.

Because of the fundamental roles of dsRNA in initiating and maintenance of PTGS, it is not surprising that SGS3/RDR6 bodies have been identified as a common target for VSRs to suppress PTGS, e.g. the VPg of a (+) RNA virus Potyvirus [38], TRIPLE GENE BLOCK PROTEIN1 (TGBp1) of potexviruses [39], P6 of (-) RNA virus Rhabdovirus [40], p2 of *Tenuivirus* rice stripe virus (RSV) [41] and V2 protein of tomato yellow leaf curl virus [42]. In addition to the VSRs, plants also encode endogenous silencing repressor such as the *RDR1* from *Nicotiana tabacum* to target the dsRNA biogenesis step [4]. The RNA decapping process could be hijacked and activated by viruses to reduce the template for synthesis of dsRNAs thereby escaping the host PTGS immune surveillance. We expect future work to uncover VSRs from other DNA and RNA viruses that may act directly on other P body components to activate its decapping activity and downregulate PTGS for the benefit of the virus.

### Working model

**Fig 7:**
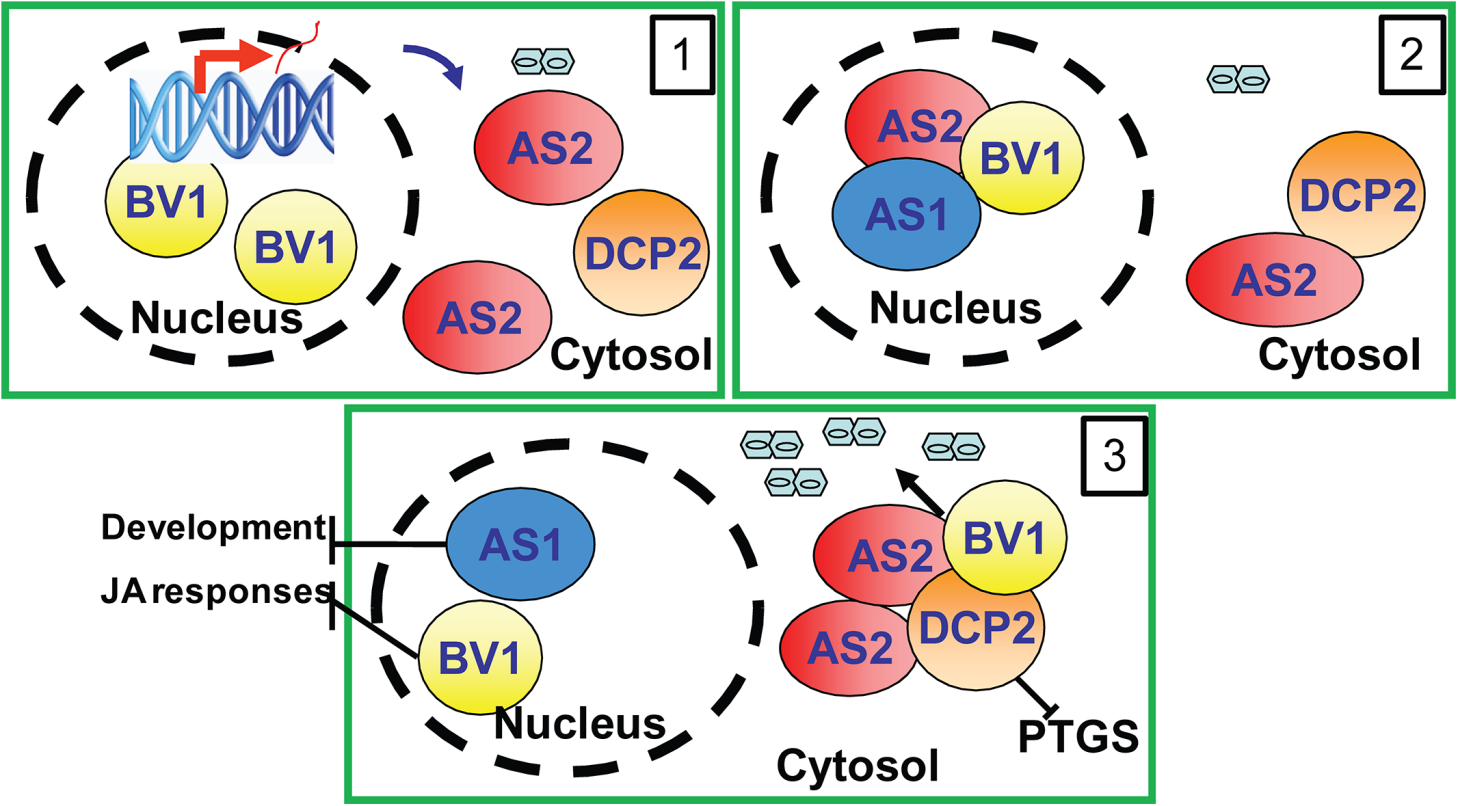
Proposed mechanism of BV1 and AS2. The viral protein BV1 causes nuclear exit of AS2 to the cytosol. Cytosolic AS2 becomes localized in P-bodies to activate decapping thereby inhibiting PTGS and facilitating virus replication.

The results in this study can be best summarized by the working model presented in Fig 7. In this model, BV1 of geminivirus translocates into the nucleus to bind and activate *AS2* expression (Frame 1). The induced AS2 protein binds to BV1 (Frame 2) which shuttles it into cytoplasmic P bodies where AS2 also promotes decapping efficiency (Frame 3). BV1 can also interact with AS2 and DCP2 to enhance RNA decapping and mRNA turnover. The accelerated aberrant RNA turnover suppresses host PTGS and make plants more sensitive to geminivirus infection and replication (Frame 3).

## Supporting Information

S1 Table DNA primers used in this study. (DOCX)

S1 Fig Re-activation of *35S*:*GUS* expression by *35S*:*BV1* in the *Arabidopsis* L1 line. The L1 line which carried a silenced *35S*:*GUS* transgene was retransformed with *35S*:*BV1*. T2 seedlings of 4 independent transgenic lines (#2, 4, 7 &11) were assayed for GUS activity. Bar = 10 mm.
(TIF)

S2 Fig The 5′ region (-2.8 to -1.2 kb) of *AS2* promoter is essential for virus-responsive expression. Red short labeling such as "*AS2p*:*GUS/*Col-0” indicates plant genetic background. Mock, *BV1*-deficient CaLCuV; CaLCuV, *BV1-*carrying CaLCuV. (A) *AS2p*:*GUS* transgene activity in WT (Col-0) seedlings. (B) *AS2p*:*GUS* transgene activity in *as2-1* mutant seedlings. (C) Left, *AS2p*:*GUS* transgenic WT plant infected with *BV1*-deficient CaLCuV; Right, *AS2p*:*GUS* transgenic WT plant infected with CaLCuV. (D) *AS2p*:*GUS* activity in transgenic WT plant infected with CaLCuV. (E) *AS2p*:*GUS* activity in transgenic *as2-1* mutant plant infected with CaLCuV. (F) *-1*.*2AS2p*:*GUS* activity in transgenic WT plants infected with CaLCuV. Bar = 10 mm.
(TIF)

S3 Fig Molecular analysis of CaLCuV-infected Col-0 and *as2-1* plants. (A) Phenotypes of Col-0, *as1-1* and *as2-1* plants. Bar = 10 mm. (B) Southern blot to detect genomic DNA of CaLCuV-infected plants (4 weeks after inoculation). (C) Small RNA Northern blot to detect viral-specific siRNAs in CaLCuV-infected WT or *as2-1* plant (4 weeks after inoculation).
(TIF)

S4 Fig *N*. *benthamiana* plants over-expressing AS2 are highly sensitive to geminivirus infection. (A-E) WT and *35S*:*AS2* transgenic *N*. *benthamiana* plants infected with a bipartite geminivirus *Indian Cassava Mosaic virus* (ICMV-Dha) at 10 dpi. Bar = 10mm. (F) and (G) Relative virus titer of WT and *35S*:*AS2* transgenic *N*. *benthamiana* plants infected with the ICMV-Dha. *NbEF1α* was served as an internal plant genomic DNA control. Values are mean ± SD (n = 3 biological replicates).
(TIF)

S5 Fig AS2, BV1 and DCP2 proteins have weak silencing suppressor activity in *N*. *benthamiana*. (A) Local silencing suppressor activity of P19 (Tomato bushy stunt virus), DCP2, and BV1. *N*. *benthamiana* (line 16c) leaves were co-infiltrated with *35S*:*GFP* along with *35S*:*P19*, or 35S:*DCP2*, or 35S:*BV1*. Bar = 10 mm. Plants of *N*. *benthamiana* line 16c agroinfiltrated with Agrobacterium strains carrying the indicated construct and inoculated plants were photographed with a yellow filter under a long-wave UV lamp at 3 dpi. (B) Western blot analysis showing GFP protein levels in *N*. *benthamiana* leaf (line 16c) infiltrated with *35S*:*GFP* along with *35S*:*BV1*, *35S*:*DCP2* or mock treatment (upper panel). WT *N*. *benthamiana* was used as a negative control. Coomassie blue-staining of the large subunit of ribulose 1,5-bisphosphate carboxylase/oxygenase (rbcL) is shown as a loading control (lower panel). (C) Systemic silencing suppressor activity of P19, DCP2, and BV1. *N*. *benthamiana* (line 16c) leaves were co-infiltrated with *35S*:*GFP* along with *35S*:*P19*, or 35S:*DCP2*, or 35S:*BV1*. Bar = 10 mm. Photos were taken at 7 days post inoculation (dpi). White arrows indicate the systemic silencing. Red color leaves/Red wing (white arrows with tail) were also observed on local leaves of 35S:*DCP2* or 35S:*BV1* co-infiltrated with *35S*:*GFP*, though the signals were weaker than those of empty control (mock) treated plants. (D) Upper panel: Northern blot analysis of *GFP*-specific siRNA levels on local leaves of 7 dpi. rRNA and tRNA levels in lower panel were used as a loading control.
(TIF)

S6 Fig Sub-cellular localization of AS2 and its mutants in transgenic *Arabidopsis* root. Roots of T3 generation transgenic *Arabidopsis* plants carrying the indicated transgene were analyzed under confocal microcopy. Bar = 100 μm. *AS2* native promoter (-2.8Kb to the translation start codon, *AS2p*:*EGFP*) was used to express EGFP as a control. *AS2* or its variants (*AS2NLS*/*AS2NES*) was fused with *EGFP* and expressed from the *AS2* native promoter.
(TIF)

S7 Fig Sub-cellular localization of AS2 and its mutants by transient expression in *N*. *benthamiana* leaf cells. Bar = 50 μm.
(TIF)

S8 Fig Amino acid sequence alignment for *Arabidopsis* AS2 and AS2 homologs from rice (OsAS2-like, Os01g0889400) and from maize (AY940681). * indicate the conserved amino acid Ile (I88 for AtAS2).
(TIF)

S9 Fig Similar activity between AS2 and AS2 mutants in promoting DCP2 decapping activity *in vitro*. Equal amount (0.5 pmol) of each of the indicated protein was added for decapping activity assay.
(TIF)

S10 Fig Relative expression level of *AtEXPL1* in *as2-1* and Col-0 WT plants. (TIF)
