# Supplementary material for: Geminivirus Activates ASYMMETRIC LEAVES 2 to Accelerate Cytoplasmic DCP2-Mediated mRNA Turnover and Weakens RNA Silencing in Arabidopsis
Source: PLoS Pathog. 2015 Oct 2;11(10):e1005196. doi: 10.1371/journal.ppat.1005196 (PMC4592220; doi:10.1371/journal.ppat.1005196)
Supplement: S1 Table — (DOCX) [file ppat.1005196.s001.docx]

**Table S1 DNA primers used in this study.**

| **Primer** | **Sequence (5’-3’)** | **Purpose** |
| --- | --- | --- |
| ASL3-F | ATACAGTCGACATGCCCAAGAGAGAAACAAAGAAG | Protein expression |
| ASL3-R | TTATTTCTAGAACCCCTCCGACCACCATAAGCCG | Protein expression |
| AS2 pro-F | ATAACAAGCTTAATGATCGGTGAGAGGGATT | Promoter cloning |
| AS2 pro-R | ATACCCTGCAGAATGATCGGTGAGAGGGATT | Promoter cloning |
| AS2-I88A F | TACGGCTGCGTCGGCGTCGCCTCTCTCCTCCAACATCAG | Point mutation |
| AS2-I88A R | CTGATGTTGGAGGAGAGAGGCGACGCCGACGCAGCCGTA | Point mutation |
| AS2-Sal5 | ATACAGTCGACATGGCATCTTCTTCAACAAACTCA | Protein expression |
| AS2-Xb3 | TTATTTCTAGACGGATCAACAGTACGGCG | Protein expression |
| AS2-NES-Bg3 | TTAGATCTGTCGAGGGTGAGACGCTCGAGAGGAGGGAGCTGGAGAGACGGATCAACAGTA | Protein expression |
| AS2NLS-Bg3 | CTCTAGATCTAACTTTGCGTTTCTTTTTCGGAGACGGATCAACAGTACG | Protein expression |
| AS2-BH5 | ATACAGGATCCATGGCATCTTCTTCAACAAACTCA | Protein expression |
| AS2-stop-Xh3 | GACGTCTCGAGTCAAGACGGATCAACAGTACGGCG | Protein expression |
| CaBV1-Xb5 | TCTAGAAAAACCATGTATCCTACAAAGTTTAGGCGTGGG | Protein expression |
| CaBV1-Xh3 | ATCCCTCGAGTCATTAACCTAAATAATCAAGATCGTAAG | Protein expression |
| CaBV1-BH3 no stop | ATCCGGATCCGGACCTAAATAATCAAGATCGTAAG | Protein expression |
| CaBV1-BH3 stop | ATCCGGATCCTCATTAACCTAAATAATCAAGATCGTAAG | Protein expression |
| Actin2-F | CGTTTCGCTTTCCTTAGTGTTAGCT | ChIP-QPCR |
| Actin2-R | AGCGAACGGATCTAGAGACTCACCTTG | ChIP-QPCR |
| Region 1-F | TGGGAACGATGTGTCTATATTG | ChIP-QPCR |
| Region 1-R | ATCTGATGTAGGCTTATTGCA | ChIP-QPCR |
| Region 2-F | GTCGGGTTTTTATTTCGGGT | ChIP-QPCR |
| Region 2-R | CGGACTTAGGAATGAGGACG | ChIP-QPCR |
| Region 3-F | GTTGACAGGCTGCGGTAAAT | ChIP-QPCR |
| Region 3-R | AACAGTTTGTGAAGGGTGGC | ChIP-QPCR |
| Region 4-F | CAGTTGAGAGATTATGTAAGCA | ChIP-QPCR |
| Region 4-R | GAGTACATTATCACAATCCTC | ChIP-QPCR |
| Region 5-F | GACACTTGCATGATCCTAAT | ChIP-QPCR |
| Region 5-R | TGATAATGAAGTTACCGTCCT | ChIP-QPCR |
| AtFAC1-R-16G | CCCCCCCCCCCCCCCCGAGTGACACAATCAAAATCTCTG | In vitro transcription |
| AtFAC1-T7-F | TAATACGACTCACTATAGGGCCTATAAATAACAACTCAA | In vitro transcription |
| EXPL1-F | GCTATCCAGTTCAGGTTCGTAGT | Q-PCR |
| EXPL1-R | AGTTCCAGATGTGAGCATCGCAA | Q-PCR |
| AS1-F | AGACAGTTCGGTCCGAGAGA | Q-PCR |
| AS1-R | ACCACTTCCCTAACCGCTTT | Q-PCR |
| AS2-F | CTCAACGAGCTTCACCCTTC | Q-PCR |
| AS2-R | TGACGAAGCTGATGTTGGAG | Q-PCR |
| BP-F | TGTTGTTTCCACATATGAGCTCTCT | Q-PCR |
| BP-R | TCATGATCAGATCGGAAGCAAT | Q-PCR |
| KNAT2-F | TTCCGCTCGACGGAAGAC | Q-PCR |
| KNAT2-R | AATCGGACGGCATCATCAAC | Q-PCR |
| KAN2-F | AAGGAACTAGATGGAAAGTGCTCAA | Q-PCR |
| KAN2-R | GCTTGTTCCCGAGATGCTTG | Q-PCR |
| Tub2-F | TCAAGAGGTTCTCAGCAGTA | Q-PCR |
| Tub2-R | TCACCTTCTTCATCCGCAGTT | Q-PCR |
| CalCuV-A-F | CCTTCTGGGGTGAGTGATGT | Q-PCR |
| CalCuV-A-R | AAACCCTGTCGTGGTCAGTC | Q-PCR |
| CalCuV-B-F | TACAAAGTTTAGGCGTGGGG | Q-PCR |
| CalCuV-B-R | TGAAAGGGCTGAGTTATGGG | Q-PCR |
| ICMV-A-F | AAGCGCTCTAATTTGGGAAAGTGCC | Q-PCR |
| ICMV-A-R | AAATGTTTGAGTACCGATTGAGGAG | Q-PCR |
| ICMV-B-F | TCTCAATTTTGGGAGATCGG | Q-PCR |
| ICMV-B-R | AAGTGGACTCCGTTGACCAC | Q-PCR |
| TRV1-RT | CATCACTTTAAAAGTACTTACTCAC | RT-PCR |
| TRV1-F | CTGGAAGGTGGGTTGTATTTCT | Q-PCR |
| TRV1-R | TAATCCGAACTGTTCAGTTCTT | Q-PCR |
