# Supplementary figures and images for: Geminivirus Activates ASYMMETRIC LEAVES 2 to Accelerate Cytoplasmic DCP2-Mediated mRNA Turnover and Weakens RNA Silencing in Arabidopsis
Source: PLoS Pathog. 2015 Oct 2;11(10):e1005196. doi: 10.1371/journal.ppat.1005196 (PMC4592220; doi:10.1371/journal.ppat.1005196)

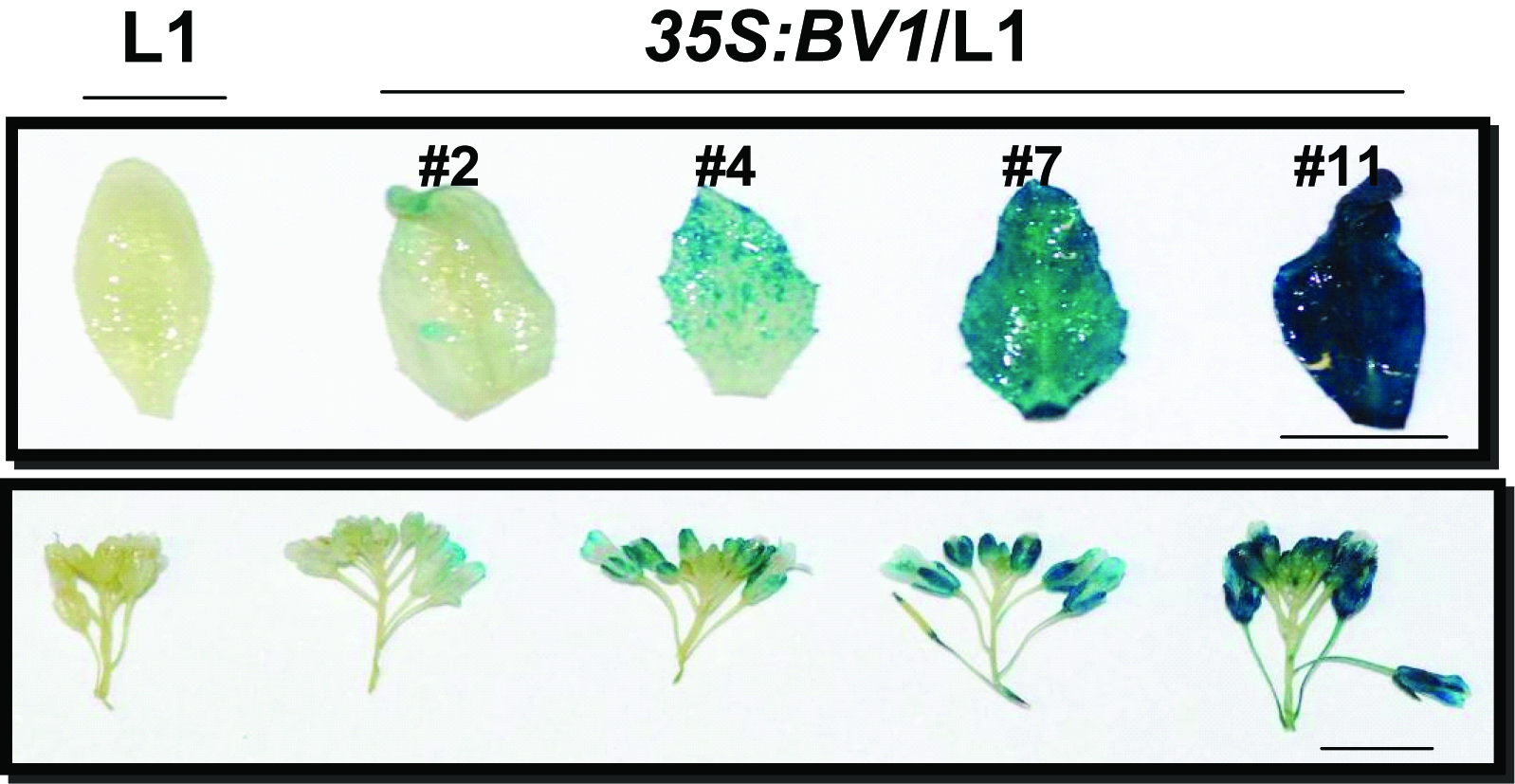

Supplement: S1 Fig — The L1 line which carried a silenced 35S:GUS transgene was retransformed with 35S:BV1. T2 seedlings of 4 independent transgenic lines (#2, 4, 7 &11) were assayed for GUS activity. Bar = 10 mm. (TIF) [file ppat.1005196.s002.tif]

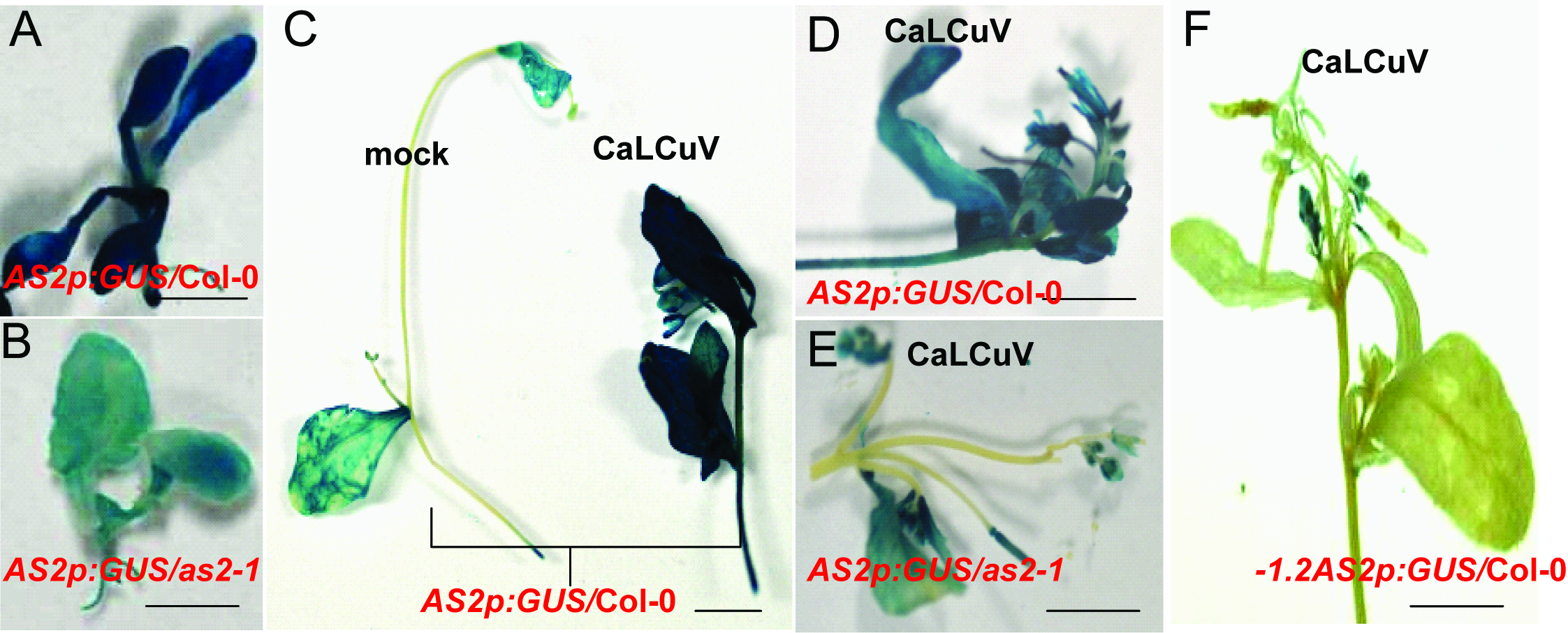

Supplement: S2 Fig — Red short labeling such as "AS2p:GUS/Col-0” indicates plant genetic background. Mock, BV1-deficient CaLCuV; CaLCuV, BV1-carrying CaLCuV. (A) AS2p:GUS transgene activity in WT (Col-0) seedlings. (B) AS2p:GUS transgene activity in as2-1 mutant seedlings. (C) Left, AS2p:GUS transgenic WT plant infected with BV1-deficient CaLCuV; Right, AS2p:GUS transgenic WT plant infected with CaLCuV. (D) AS2p:GUS activity in transgenic WT plant infected with CaLCuV. (E) AS2p:GUS activity in transgenic as2-1 mutant plant infected with CaLCuV. (F) -1.2AS2p:GUS activity in transgenic WT plants infected with CaLCuV. Bar = 10 mm. (TIF) [file ppat.1005196.s003.tif]

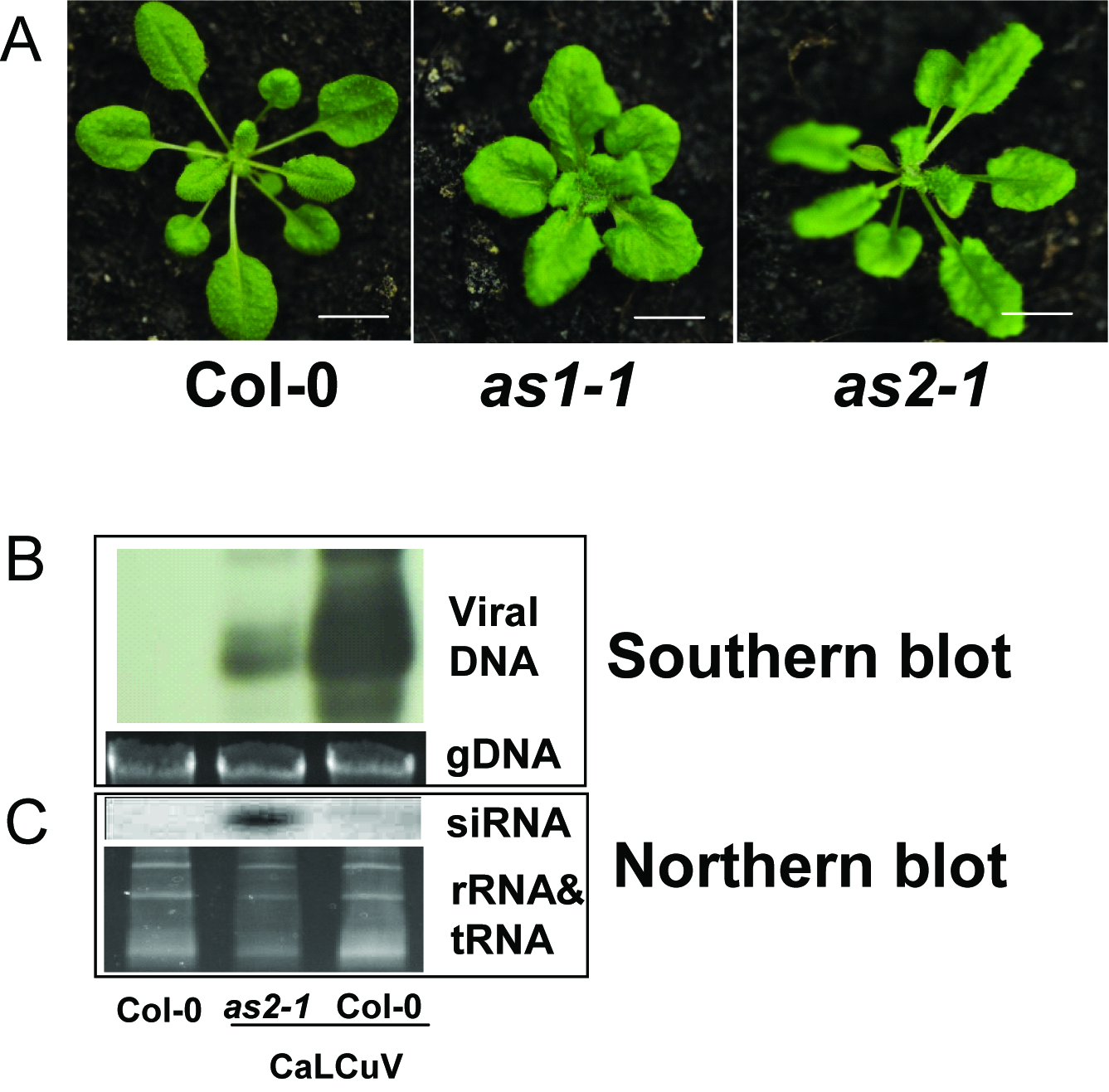

Supplement: S3 Fig — (A) Phenotypes of Col-0, as1-1 and as2-1 plants. Bar = 10 mm. (B) Southern blot to detect genomic DNA of CaLCuV-infected plants (4 weeks after inoculation). (C) Small RNA Northern blot to detect viral-specific siRNAs in CaLCuV-infected WT or as2-1 plant (4 weeks after inoculation). (TIF) [file ppat.1005196.s004.tif]

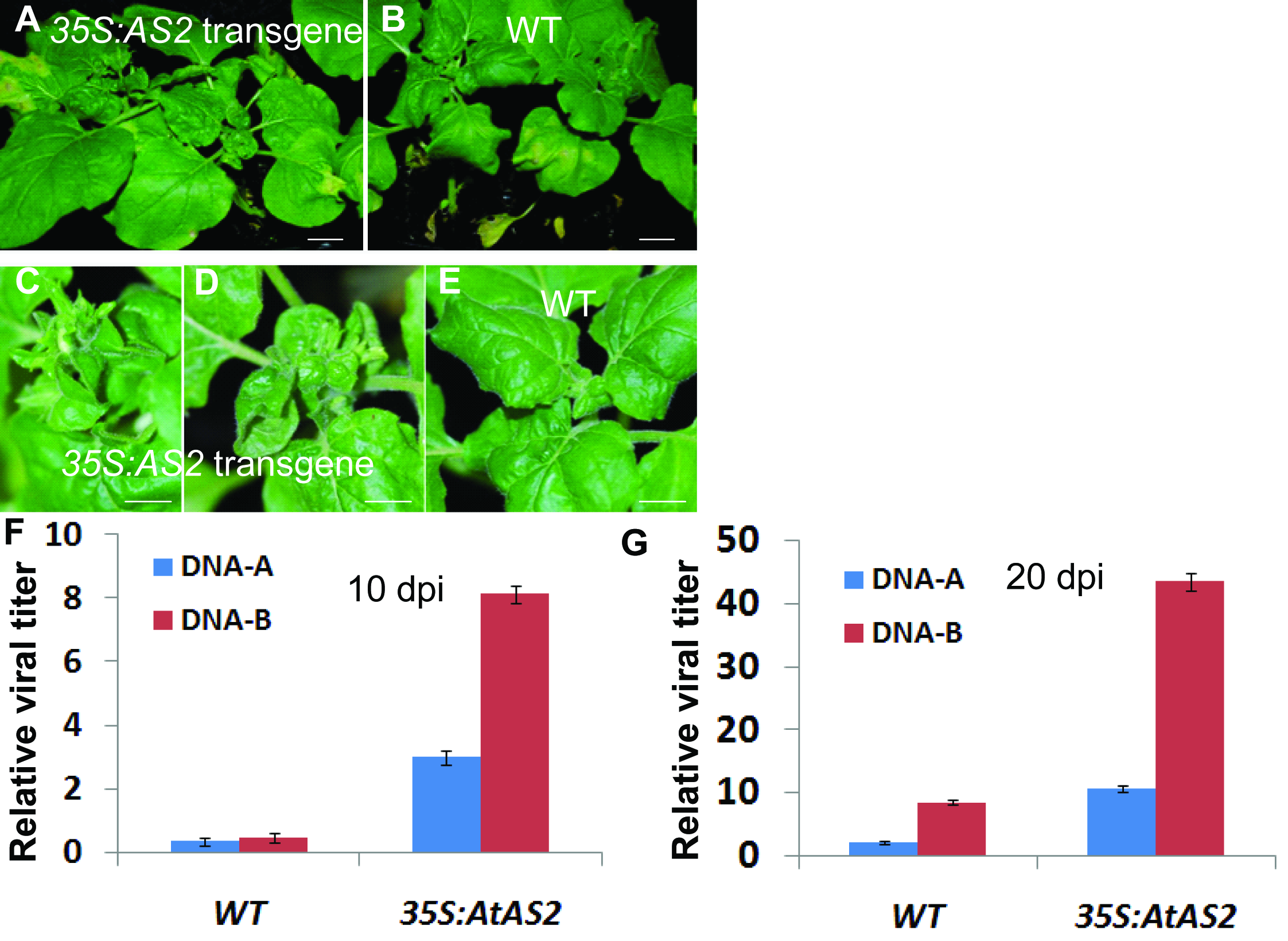

Supplement: S4 Fig — (A-E) WT and 35S:AS2 transgenic N. benthamiana plants infected with a bipartite geminivirus Indian Cassava Mosaic virus (ICMV-Dha) at 10 dpi. Bar = 10mm. (F) and (G) Relative virus titer of WT and 35S:AS2 transgenic N. benthamiana plants infected with the ICMV-Dha. NbEF1α was served as an internal plant genomic DNA control. Values are mean ± SD (n = 3 biological replicates). (TIF) [file ppat.1005196.s005.tif]

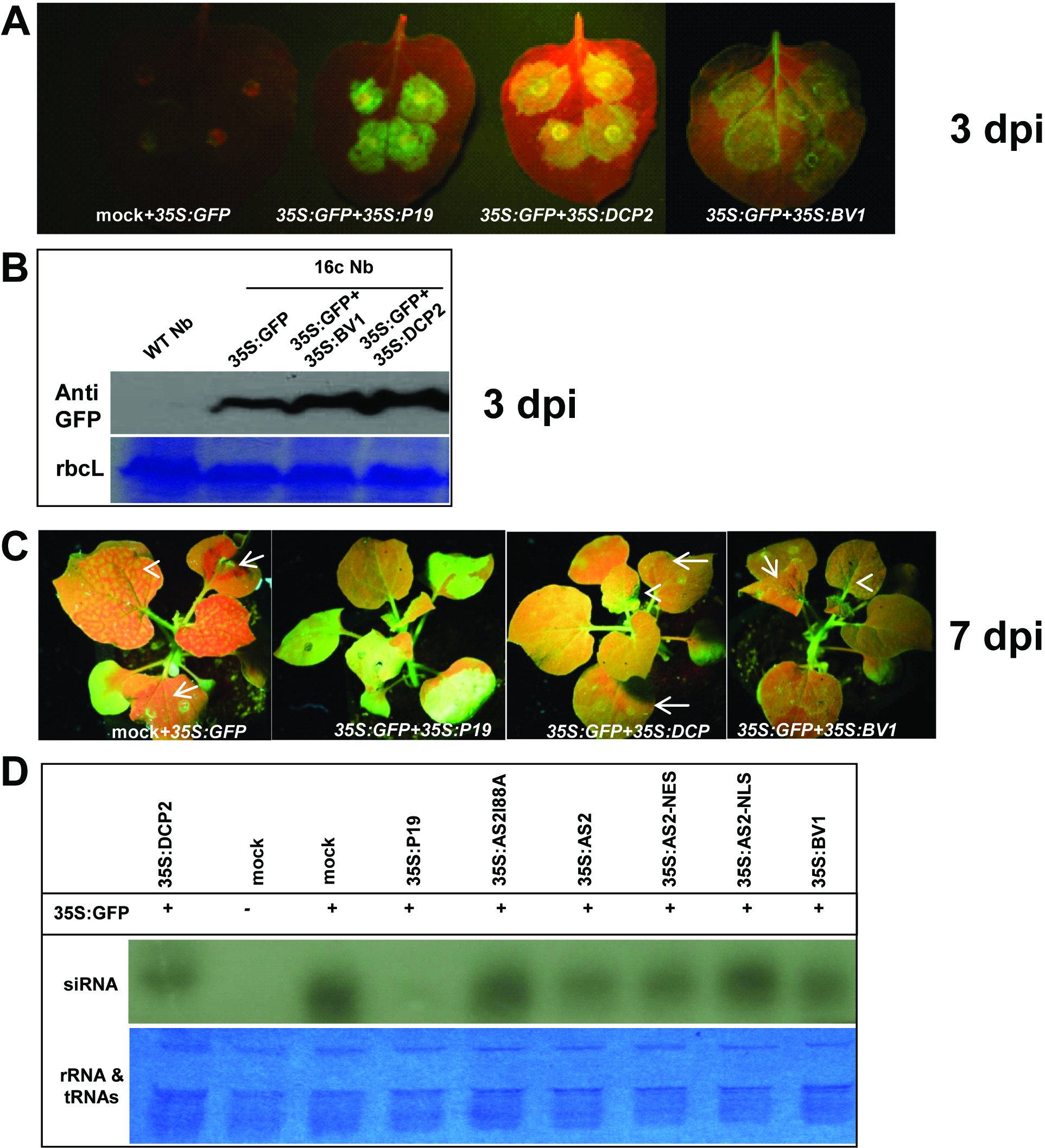

Supplement: S5 Fig — (A) Local silencing suppressor activity of P19 (Tomato bushy stunt virus), DCP2, and BV1. N. benthamiana (line 16c) leaves were co-infiltrated with 35S:GFP along with 35S:P19, or 35S:DCP2, or 35S:BV1. Bar = 10 mm. Plants of N. benthamiana line 16c agroinfiltrated with Agrobacterium strains carrying the indicated construct and inoculated plants were photographed with a yellow filter under a long-wave UV lamp at 3 dpi. (B) Western blot analysis showing GFP protein levels in N. benthamiana leaf (line 16c) infiltrated with 35S:GFP along with 35S:BV1, 35S:DCP2 or mock treatment (upper panel). WT N. benthamiana was used as a negative control. Coomassie blue-staining of the large subunit of ribulose 1,5-bisphosphate carboxylase/oxygenase (rbcL) is shown as a loading control (lower panel). (C) Systemic silencing suppressor activity of P19, DCP2, and BV1. N. benthamiana (line 16c) leaves were co-infiltrated with 35S:GFP along with 35S:P19, or 35S:DCP2, or 35S:BV1. Bar = 10 mm. Photos were taken at 7 days post inoculation (dpi). White arrows indicate the systemic silencing. Red color leaves/Red wing (white arrows with tail) were also observed on local leaves of 35S:DCP2 or 35S:BV1 co-infiltrated with 35S:GFP, though the signals were weaker than those of empty control (mock) treated plants. (D) Upper panel: Northern blot analysis of GFP-specific siRNA levels on local leaves of 7 dpi. rRNA and tRNA levels in lower panel were used as a loading control. (TIF) [file ppat.1005196.s006.tif]

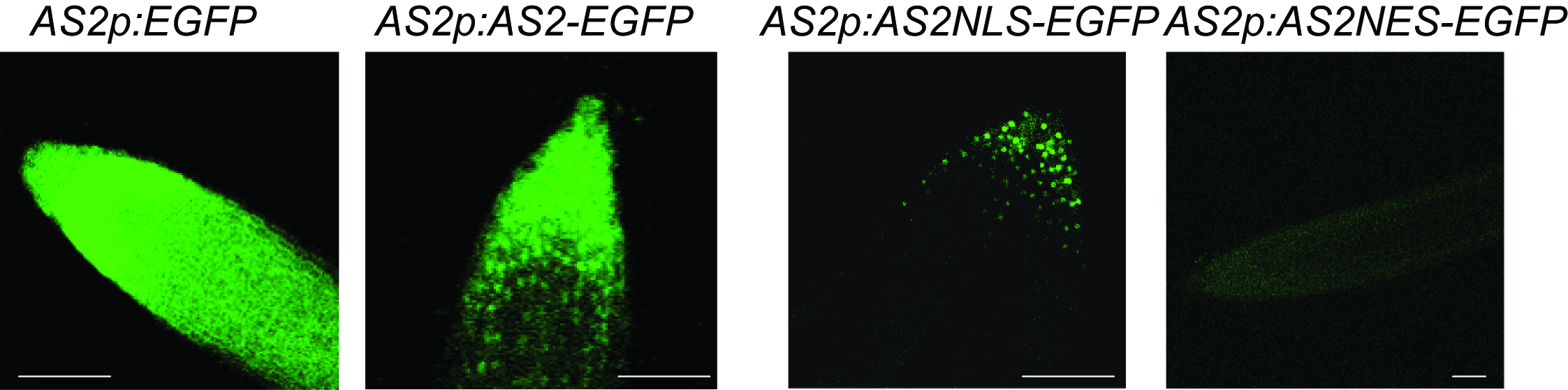

Supplement: S6 Fig — Roots of T3 generation transgenic Arabidopsis plants carrying the indicated transgene were analyzed under confocal microcopy. Bar = 100 μm. AS2 native promoter (-2.8Kb to the translation start codon, AS2p:EGFP) was used to express EGFP as a control. AS2 or its variants (AS2NLS/AS2NES) was fused with EGFP and expressed from the AS2 native promoter. (TIF) [file ppat.1005196.s007.tif]

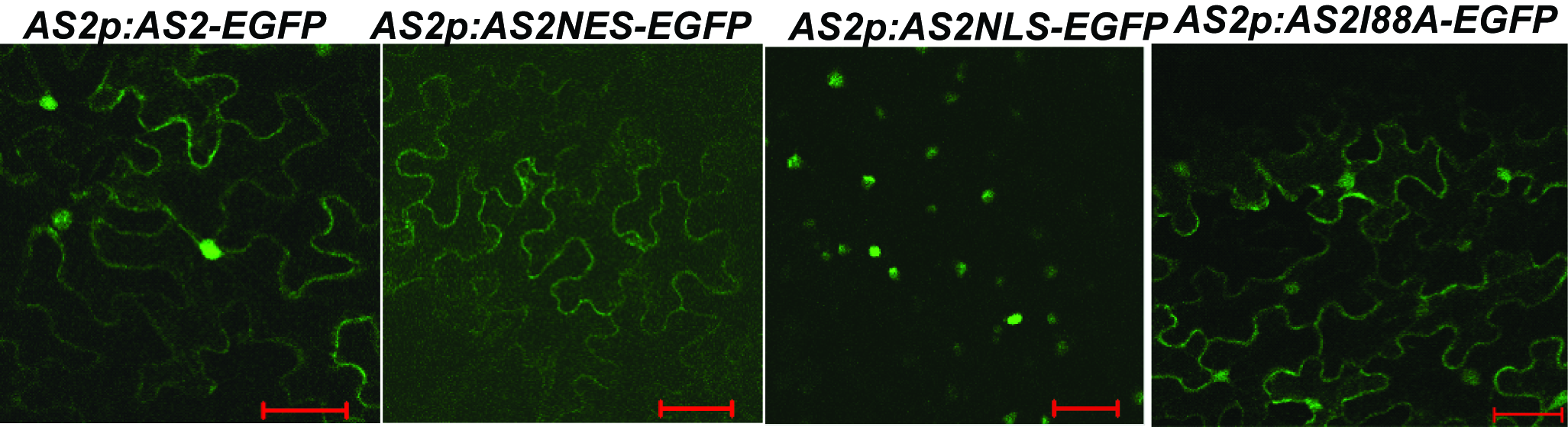

Supplement: S7 Fig — Bar = 50 μm. (TIF) [file ppat.1005196.s008.tif]

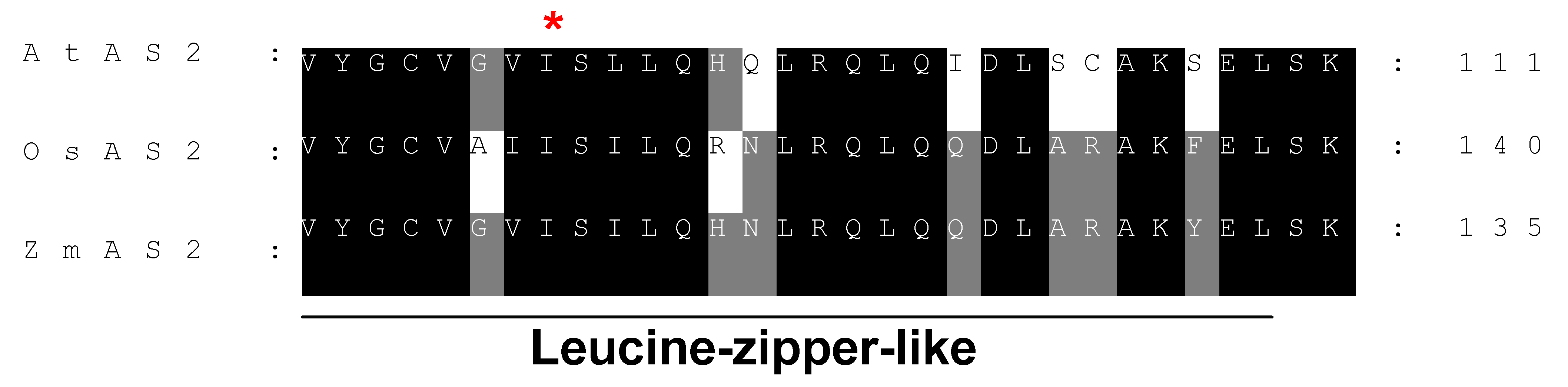

Supplement: S8 Fig — * indicate the conserved amino acid Ile (I88 for AtAS2). (TIF) [file ppat.1005196.s009.tif]

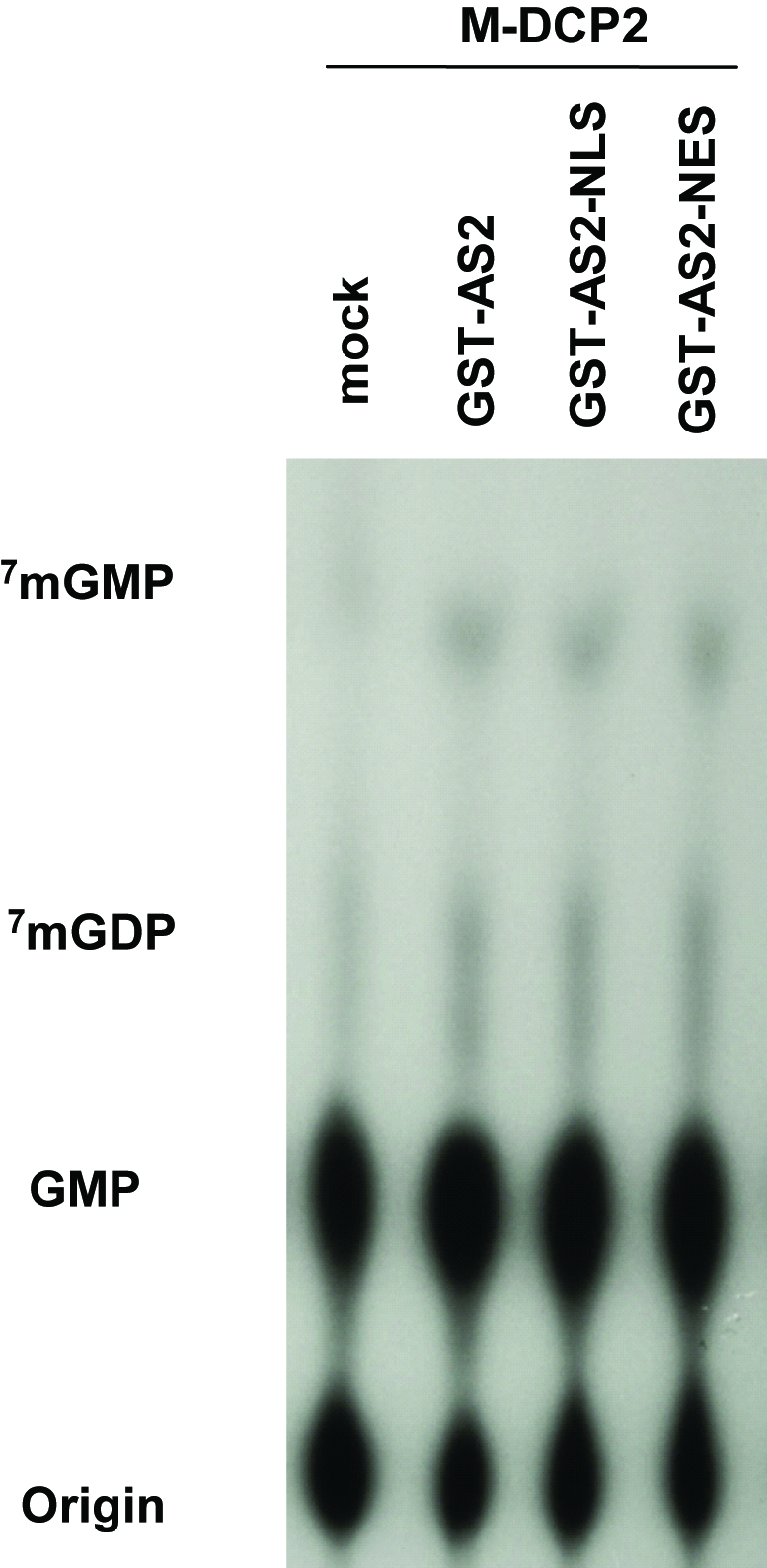

Supplement: S9 Fig — Equal amount (0.5 pmol) of each of the indicated protein was added for decapping activity assay. (TIF) [file ppat.1005196.s010.tif]

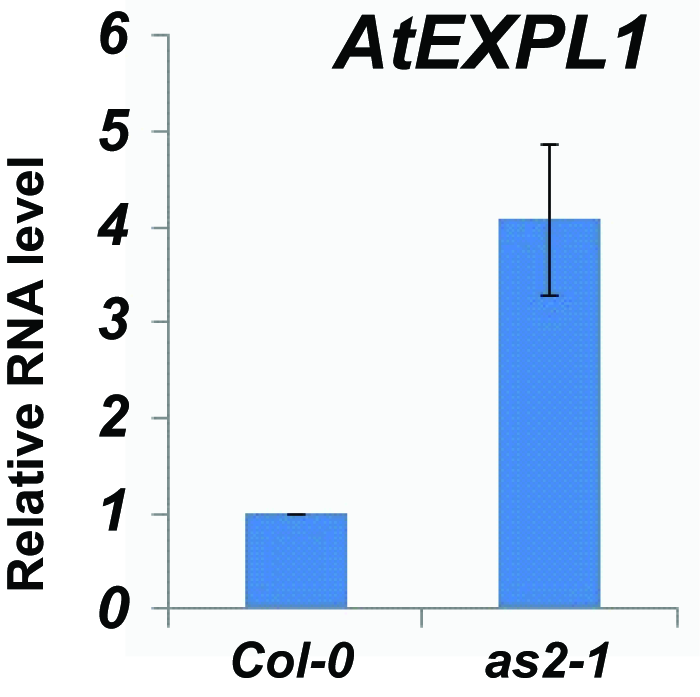

Supplement: S10 Fig — (TIF) [file ppat.1005196.s011.tif]
